# Stress responsiveness in a wild primate predicts survival across an extreme El Niño drought

**DOI:** 10.1126/sciadv.adq5020

**Published:** 2025-01-22

**Authors:** Sofia C. Carrera, Irene Godoy, Colleen M. Gault, Ashley Mensing, Juliane Damm, Susan E. Perry, Jacinta C. Beehner

**Affiliations:** ^1^ Department of Psychology, University of Michigan, Ann Arbor, MI 48109, USA.; ^2^ Lomas Barbudal Monkey Project, Lomas Barbudal Biological Reserve, Bagaces, Costa Rica.; ^3^ Department of Animal Behaviour, University of Bielefeld, Bielefeld 33501, Germany.; ^4^ Department of Anthropology, University of California, Los Angeles, CA 90095, USA.; ^5^ Instituto de Neuroetología, Universidad Veracruzana, Xalapa 91190, Mexico.; ^6^ Department of Anthropology, University of Michigan, Ann Arbor, MI 48109, USA.

## Abstract

We know more about the costs of chronic stress than the benefits of the acute stress response—an adaptive response that buffers organisms from life-threatening challenges. As yet, no primate study has empirically identified how the stress response adaptively affects evolutionary fitness. Here, we take advantage of a natural experiment—an El Niño drought—that produced unprecedented mortality for wild white-faced capuchins. Using a reaction norm approach, we provide evidence from primates that a more robust stress response to a challenge, measured using fecal glucocorticoids, predicts a greater likelihood of survival. We show that individuals with greater stress responsiveness to previous droughts later had higher survival across a severe El Niño drought. Evolutionary models need empirical data on how stress responsivity varies in adaptive ways. While we cannot buffer subjects from catastrophic events, we can use them to understand which aspects of the stress response help animals to “weather the storm.”

## Teaser

White-faced capuchins with a stronger stress response to previous droughts were more likely to survive a severe El Niño drought.

## INTRODUCTION

Nearly 90 years after H. Selye introduced the term “stress” to the biomedical world (*1*), we still have a limited understanding of how the vertebrate stress response affects evolutionary fitness (*2*). There have been two primary avenues for stress research. In the biomedical field, research has focused on how chronic stressors adversely affect human health and longevity—dominating the literature with human examples of how a chronic stress response [i.e., prolonged activation of the hypothalamic-adrenal-pituitary (HPA) axis in response to frequent or long-lasting stressors] can lead to anxiety, depression, and systemic disease (*3*–*6*). This approach has been enormously successful at improving human health and well-being by identifying risk factors and prevention strategies for people with stressful lives (*7*).

By contrast, in the field of behavioral ecology and evolution, research has focused primarily on how the acute stress response adaptively enhances evolutionary fitness via individual survival or reproduction. This approach has not had nearly the same level of empirical success. Because the mechanisms that regulate endocrine systems are enormously complex (e.g., plasticity of the endocrine response, magnitude of the response, receptor number and function, and speed and valence of feedback loops) and because the functional outcomes are empirically difficult to measure, evolutionary research has yet to identify a set of unifying principles about what is (and is not) an adaptive endocrine response (*2*).

Despite uncertainty about which aspects of HPA regulation are under selection, there is some consensus that, in the face of a challenge, an adaptive stress response (i.e., a fitness-enhancing one) should be characterized by low baseline glucocorticoids, a rapid increase following the onset of a stressor, followed quickly by a return to baseline after the challenge is over (*8*). We use “stress response” to refer to activation of the HPA axis in response to a variety of physiological challenges that increase the secretion of glucocorticoids—steroid hormones produced by the adrenal glands that regulate metabolism, immune function, and the stress response in vertebrates (*6*). Thus, an adaptive HPA response should permit and prepare the internal defense mechanisms needed to survive a challenge (e.g., mobilizing glucose for quick energy).

However, even an adaptive stress response has trade-offs. Frequent or prolonged activation of the HPA axis takes a cumulative toll on an organism. This “wear-and-tear” on an organism is known as allostatic load (*9*). Organisms experiencing more energetic challenges (e.g., frequent predator attacks and exposure to pathogens) are forced to allocate more energy to dealing with (and recovering from) these anabolic challenges. All things being equal, these individuals have less energy in their overall budget to allocate to catabolic processes unrelated to immediate survival (but nevertheless important for overall fitness) such as growth and reproduction (*10*). Across a lifetime, a higher allostatic load can shorten an organism’s lifespan. For example, in wild yellow baboons, females with the highest HPA axis activation across their adult lives died more than 5 years earlier than females with the lowest activation (*11*). While such wildlife examples externally validate our understanding that sustained activation of the HPA axis is associated with reduced lifespans, they are unable to help us understand how the stress response adaptively buffers organisms from life-threatening challenges. Accomplishing this latter task has proven elusive—particularly for primates in natural populations (*12*). To date, no study has demonstrated the adaptive nature of the stress response in wild primates (*12*, *13*).

Because most research on wild primate populations is based on opportunistic hormone sampling with weekly to monthly gaps between samples, primate studies are typically restricted to a between-subjects approach. This approach compares glucocorticoid profiles of individuals experiencing different levels of stressors and then correlates them with fitness outcomes (*14*). Because individuals experiencing many stressors simultaneously have higher glucocorticoids and poorer fitness outcomes, this can produce a spurious relationship between high glucocorticoids and lower fitness. This correlational relationship, known as the Cort-Fitness Hypothesis, can be misleading [as recognized by others (*8*)] when the assumption is that the increase in cortisol (or glucocorticoids) causes the reduction in fitness. To remedy this, many have advocated for an individual-level, norm-of-reaction approach that identifies whether one individual’s response to a stressor is associated with a more favorable outcome than another’s response to the same stressor (*15*). That is, what is each individual’s glucocorticoid change in response to the same stressor, and do some responses predict survival more than others?

These are not easy data to collect in wild primates. First, individual primates rarely experience identical stressors—either in their magnitude or in their frequency. For example, different primate populations may have variation in the threat posed by predators. For primate populations with higher predation pressure, it then becomes difficult to decipher whether lower fitness results directly from the dangers that accompany those predators or indirectly from the chronic stress caused by constant vigilance. The problem, therefore, is finding a situation in wild primates where a stressor of the same (or similar) magnitude impacts all individuals evenly.

Second, it is difficult to quantify the benefits of a “successful” stress response (i.e., one that allows an individual to survive the challenge). To measure the benefits of variable stress responses in a natural system, we must ask a counterfactual question: How would the same (or similar) individual’s fitness have differed if it had shown a different response to the same (or similar) stressor? To even approach an answer to this question, we need to identify a singular severe stressor in a population, measure each individual’s stress response to this stressor, and track individual fitness outcomes across the event—each outcome being a probabilistic reduction in risk of death (or an increase in reproduction, depending on which fitness component is the focus).

Measuring the impact of a probabilistic outcome is not an easy dataset to generate. Where experimentation is possible and ethical, researchers can induce a stressor in a population with already-known endocrine profiles and observe the outcomes in natural settings (*13*, *16*). However, such experiments generally do not impose challenges severe enough to kill their study subjects, and most of these studies are conducted on small, short-lived species that are phylogenetically more distant from humans than primates (*13*). Because experimentation is not generally an option for stress research in wild primates [but see (*17*)], the only way to ethically apply this experimental approach is to take advantage of a natural disaster that universally strikes all individuals in a population.

**Fig. 1.**
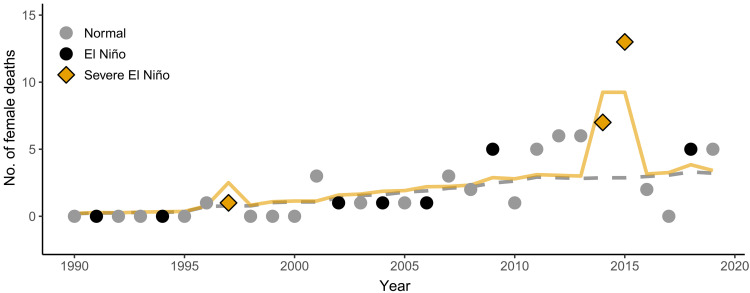
Adult female mortality spikes during severe El Niño events. Female deaths across years for the wild population of white-faced capuchins at Lomas, which experienced 3.3 times higher rates of adult female mortality during the 2014–2016 El Niño drought compared to non–El Niño years (*z* = 4.19, *P* < 0.001). Points denote the number of deaths in each year (gray circle, normal years; black circle, El Niño years; yellow diamond, severe El Niño years). Exposure (i.e., approximate number of adult females available to die) in each year is in gray italics just above the *x* axis. The gray dashed line indicates the expected number of deaths for each year on the basis of the exposure and the (low) average rate of death across all years (0.049). The yellow line shows the binomial model predictions for female deaths in each year.

We take advantage of one such natural experiment, when an El Niño–related drought resulted in extremely low rainfall across Central America over a 2-year period (May 2014 to May 2016), with particularly devastating effects on the tropical dry forests of Costa Rica (*18*). This El Niño drought was one of the top three most-severe El Niño droughts in recorded history (*18*) and coincided with unprecedented levels of female mortality in a wild population of white-faced capuchin monkeys (*Cebus imitator*, formerly *Cebus capucinus*) from the Reserva Biológica Lomas Barbudal of Costa Rica (Fig. 1). This catastrophe provided us with a unique natural experiment for investigating the fitness consequences of HPA axis activation in wild primates.

To measure HPA activity, we used fecal glucocorticoid metabolite concentrations (hereafter, “glucocorticoids”) collected repeatedly from individuals in the 6 years before the El Niño drought (2008 to 2013). Because fecal hormones are noninvasive and provide an integrated hormone measurement over several hours (*19*, *20*), this is the preferred method for quantifying HPA axis activity in wild populations. In addition, rather than using static glucocorticoid metrics for subjects, we incorporated changes in glucocorticoids across challenges, allowing us to identify endocrine flexibility in response to unpredictable, drought conditions.

To avoid bias, the subjects included in our analyses were selected before glucocorticoid concentrations were measured. Nineteen adult females died during the El Niño drought. Of these 19 deaths, we included as study subjects the 14 females with sufficient hormone samples collected in the years leading up to the drought (“fatalities”) for the analysis of glucocorticoids (*21*). Then, to achieve a balanced dataset, we matched each fatality with a surviving female from the same group (“survivors”; see Materials and Methods for details) for a total dataset of 28 females. Hormone sample exclusion criteria for these subjects are detailed in Materials and Methods.

The El Niño drought was our natural experiment. We were unfortunately unable to sample hormones from the fatalities across El Niño itself. Therefore, we used HPA activity for these females across previous, less-severe droughts, for which we had hormone samples. Our approach was to examine whether an individual’s HPA response to previous less-severe droughts (during 2008 to 2013) predicted whether they survived the severe El Niño drought (during 2014 to 2016). Specifically, we estimated each individual’s change in glucocorticoids (i.e., their reaction norm) from “nondrought” to “drought” periods, and we then used this change to predict survival. Although capuchins live in a highly seasonal environment with long dry seasons characterized by months of no rain, these predictable dry seasons were not considered droughts since little to no rain is expected as per the annual seasonal pattern (*22*). We therefore broadly defined “drought” as deficits in rainfall beyond the typical seasonal patterns of a tropical dry forest. We predicted that individuals who mounted a stronger HPA response (i.e., a steep and positive slope) to previous droughts would be more likely to survive the more-severe El Niño drought than those with a weaker response (i.e., a flatter slope). Unlike starvation circumstances (*23*), mounting a robust stress response during challenges where energy is scarce (but still available) should be adaptive (*16*) by increasing foraging intensity (*24*, *25*) or mobilizing energy reserves (*6*, *26*).

**Fig. 2.**
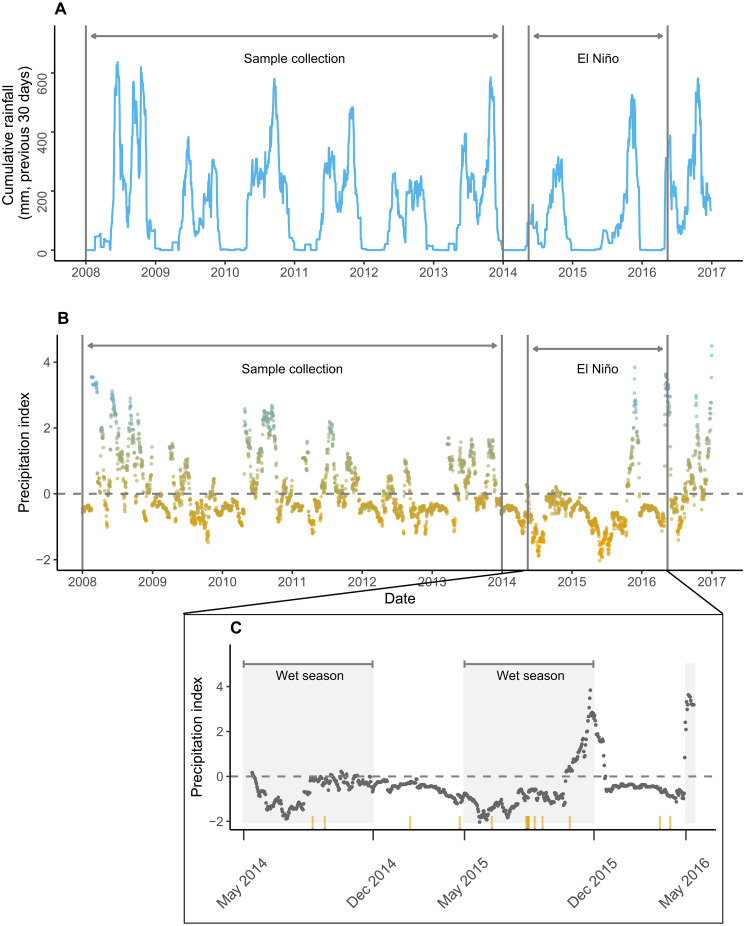
Rainfall and precipitation index across sample collection and the drought surrounding El Niño. (**A**) Cumulative 30-day rainfall from the Palo Verde research station across the sample collection period (2008–2013) and the subsequent El Niño drought (May 2014 to May 2016). (**B**) Values for our precipitation index for the same time period. Negative values indicate when rainfall was lower than expected, and positive values indicate when rainfall was higher than expected. Horizontal gray dotted line at 0 represents when rainfall was exactly as expected on the basis of the reference years (1996–2013). Warm (orange) and cool (blue) colors have been added to highlight lower and higher rainfall than expected. (**C**) The inset zooms in on El Niño years only to indicate when capuchin deaths occurred (orange tick marks on the *x* axis). Note that most deaths (10 of 14) occurred during what should have been the wet season (May 1–Nov 30).

To calculate our drought index, we first used 18 years (1996 to 2013) of daily precipitation data from the Organization for Tropical Studies Palo Verde field station to generate a precipitation index, calculated as the number of standard deviations above/below the mean for each day on the basis of the 30-day cumulative precipitation mean. This precipitation index effectively captures the severity of the El Niño drought [see the extra-low values during the El Niño period in Fig. 2 (A and B)]. We then reverse coded our precipitation index so that positive values were associated with lower-than-expected rainfall and negative values were associated with higher-than-expected rainfall (hereafter, our “drought index”).

While a dry season can have higher rainfall than is typical for that season, it rarely has lower rainfall than is typical, given the already-low seasonal expectations for the dry season [to which we expect local species to be well-adapted (*27*, *28*)]. Therefore, droughts have more disruptive potential in the wet season than in the dry season. For example, during the severe El Niño drought, the lowest points on the precipitation index were observed during what should have been the start of each wet season (Fig. 2C and fig. S1). Rather than use arbitrary dates to distinguish between the wet and dry seasons in our analyses, we calculated *z*-scores for rainfall across the year to create a continuous “expected rainfall” variable; this allowed us to specifically examine the effects of drought when rainfall was expected to be high. When rainfall adheres to seasonal patterns (e.g., wet during the wet season and not wet during the dry season), we refer to this as “seasonally appropriate rainfall.”

We expected our subjects’ HPA response during previous less-severe droughts to reflect how they responded during the El Niño drought. We maintain that rain deficits during even less-severe droughts (the lowest points during sample collection period; Fig. 2B) present a moderate (to high) ecological stressor for capuchins for two reasons. First, the dry season months at Lomas (as well as another capuchin population, Santa Rosa) are generally characterized by higher cortisol in capuchins, suggesting that lower primary productivity presents a metabolic challenge (*21*). Second, the dry season months just before the start of the wet season indicate the lowest levels of capuchins’ feeding rates on their primary foods of fruits and insects (*29*), suggesting that the expected seasonal shift to more abundant food resources in the wet season is critical for recovering from dry season deficits each year. In other words, the most severe effects of any drought should hit when dry periods extend beyond the typical dry season. As further evidence, although mortality is generally not linked to season in the Lomas population (*30*), during the severe El Niño drought of 2014 to 2016, a majority of fatalities occurred during what should have been the wet seasons (see orange vertical marks on the *x* axis; Fig. 2C).

## RESULTS

### Mortality was higher during El Niño years

Our model predicting female mortality on the basis of the occurrence of El Niño droughts was better than the null model (dAICc = 11.3), which contained no predictor variables. Mortality rate during most El Niño years did not differ from rates during normal years (when no El Niño occurred), but the mortality rate during severe El Niño droughts (1997, 2014, and 2015) was predicted to be 3.3 times higher than the rate in normal years (*z* = 4.19, *P* < 0.001; Fig. 1).

### Higher HPA responsiveness during previous droughts predicted survival during El Niño

We built a Bayesian generalized linear mixed-effects model (GLMM; family, Bernoulli) to predict the probability of surviving the El Niño event on the basis of individual-level reaction norms (reaction norms were extracted from a Bayesian LMM that examined glucocorticoids as a function of expected rainfall, drought index, and their interaction; table S1; see Materials and Methods). In this survival model (“glucocorticoids-predicting-survival model”), we included individual-level reaction norms [scaled best linear unbiased predictors (BLUPs)] as continuous predictors while controlling for the potential confounds of age and dominance rank (assigned at the start of the El Niño drought). Remember that we were specifically interested in the individual reaction norms during periods of high expected rainfall, since this allowed us to examine individual differences in reactivity to drought during what should have been wet seasons. In addition, we controlled for collection time and reproductive state (because these are known to affect glucocorticoids in this dataset) (table S2), added individual ID and group ID as random effects, and included a random slope by individual ID for the drought risk index, expected rainfall, the interaction between drought risk and expected rainfall, collection time, and reproductive state.

**Fig. 3.**
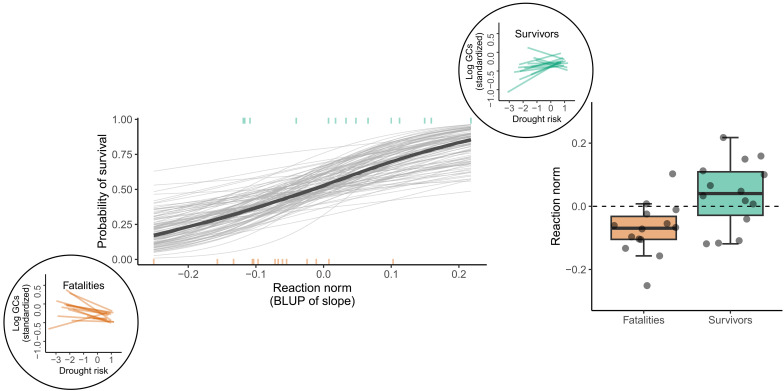
Capuchins with an increase in glucocorticoids during wet-season droughts were more likely to survive El Niño. The main figure is a spaghetti plot of linear predictions using the conditional_effects() function from “brms,” with the number of trials set to 1. Thin gray lines represent 100 random draws from the posterior distribution, and the thick black line represents the estimated population mean. The probability of survival increased as the slope of the reaction norm (BLUP of slope) increased, or in other words, individuals were more likely to survive if they were predicted to have higher glucocorticoids (GCs) when drought risk was higher. Reaction norms for each survivor are indicated by the top rug (i.e., tick marks at the top of the figure in green) and those for each fatality indicated by the bottom rug (i.e., tick marks at the bottom of the figure in orange). Figure insets in circles depict individual reaction norms for fatalities (lower left) and survivors (upper right). As the drought risk index increased (recall that the precipitation index has been inverted and positive values indicate higher drought risk), individuals that secreted more glucocorticoids (in response to increasing drought risk) were more likely to survive the severe El Niño drought than those that secreted less glucocorticoids. The inset box plot to the right compares the reaction norms (i.e., BLUPs for slope) between fatalities and survivors. Note that only 4 survivors (of 14) had negative reaction norms, and only 2 fatalities (of 14) had positive reaction norms.

Positive values for reaction norms (positive slopes) in response to increasing drought risk in the wet season predicted a greater probability of surviving the El Niño drought {β = 0.73, 89% confidence interval (CI) = [0.17, 1.28]} (Fig. 3 and Table 1). In other words, capuchins whose reaction norm was 1 SD above the population mean had a survival probability of 0.66 and were two times more likely to survive the El Niño drought compared to capuchins with reaction norms of 1 SD below the population mean (probability of 0.33). [These results are replicated in the Supplementary Materials using alternative methods (fig. S2 and table S3).] Thus, the capuchins with a more robust HPA response to droughts during what should have been wetter months—as indicated by an increase in glucocorticoids relative to the mean increase for the population—were more likely to survive the severe El Niño drought.

**Table 1.** Glucocorticoids-predicting-survival model summary and diagnostics for the Bayesian GLMM (Bernoulli) predicting survival from individual capuchins’ reaction norm BLUPs (intercept and slope). Predictor variables were scaled to a mean of 0 and a standard deviation of 1. Model output is on the logit scale.

Parameter	Estimate	Est. error	89% CI	Bulk ESS	Tail ESS	$\hat{R}$
Reaction norm slope	0.73	0.35	**[0.17, 1.28]**	4672	3135	1.00
Reaction norm intercept	0.09	0.34	[−0.44, 0.62]	4741	2677	1.00
Age	−0.44	0.34	[−0.98, 0.10]	4619	3035	1.00
Rank	0.27	0.34	[−0.26, 0.80]	4364	3095	1.00

### Survivors had higher HPA responsiveness during previous droughts

The use of BLUPs is known to be anticonservative because they are point estimates (*31*); BLUPs are predictions from a model and not true individual phenotypic data. Therefore, to confirm our results, we used an alternative modeling approach with the same dataset. For this analysis, we flipped the direction of our question, asking instead: Does individual survival during the El Niño drought predict variation in glucocorticoids across previous droughts? This analysis (“survival-predicting-glucocorticoids model”) allowed us to make use of all individual hormone samples rather than just one reaction norm estimate from each individual derived from these hormone samples.

Using another Bayesian LMM, we included the same fixed effects of collection time and reproductive state, as well as the same random effects of individual ID and group ID. Because droughts during the wet season were the most challenging, we added a three-way interaction between drought risk index, expected rainfall, and survival (in addition to the lower-level interactions). As before, we included a random slope by individual ID for the drought risk index, expected rainfall, the interaction between drought risk and expected rainfall, collection time, and reproductive state.

**Fig. 4.**
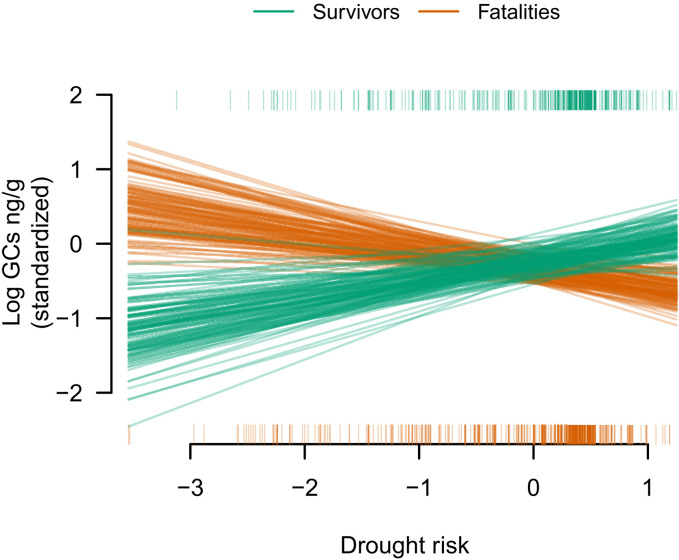
Survivors of El Niño had increasing glucocorticoids in response to increasing drought risk during the wet season. During the wet season (when droughts can have a catastrophic effect on food supply for capuchins), individuals that later survived the El Niño drought (green) exhibited increasing glucocorticoids as drought risk increased. By contrast, those that did not survive El Niño (orange) showed the opposite pattern. Green and orange lines represent 100 draws from the posterior distribution. To aid visual interpretation, drought risk on the *x* axis is an inversion of the precipitation index, such that positive values indicate higher drought risk (i.e., lower rainfall than is typical). Although expected rainfall was a continuous measure in all models, here, we show the effect of drought risk on glucocorticoids (log GCs, nanograms per gram) only during times with high expected rainfall (i.e., standardized rainfall values of +1 SD, which corresponds to the wet season). The rug plot (vertical tick marks along the *x* axes) indicates at what drought risk value each individual fecal sample was collected [top rug (green) for survivors and bottom rug (orange) for fatalities].

As expected, capuchins that survived the El Niño drought had greater glucocorticoid responses to previous droughts compared with fatalities {β = 0.23, 89% CI = [0.09, 0.36]}, and the relationship between drought risk and survival depended on expected rainfall {three-way interaction: β = 0.24, 89% CI = [0.06, 0.43]; Fig. 4 and table S4}. For example, the model predicted that survivors would have 16% higher log glucocorticoids compared to fatalities if the drought risk index was high (+1 SD) during times of high expected rainfall (standardized rainfall = +1 SD). These results are replicated in the Supplementary Materials using alternative methods (fig. S3 and table S5).

### There was no evidence for HPA dysregulation in fatalities

**Fig. 5.**
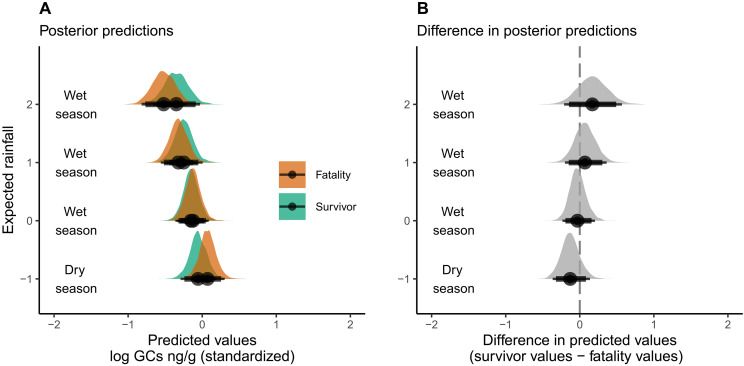
During times with seasonally appropriate rainfall, there were no differences detected between the glucocorticoids of survivors and fatalities. Posterior predictions (**A**) and the difference in posterior predictions (**B**) were generated from the survival-predicting-glucocorticoids model with values of expected rainfall set to 2 (wet season), 1 (wet season), 0 (wet season), and −1 (dry season) while holding the variable “drought risk” constant at 0 (i.e., seasonally appropriate rainfall). Half-eye density plots include posterior means (black circles), 89% probability density intervals (thick black lines), and 95% probability density intervals (thin black lines).

Results from our models revealed no evidence of HPA dysregulation for fatalities (table S4). During times with seasonally appropriate rainfall, capuchin survivors and fatalities exhibited similar glucocorticoid levels, as survival status had no main effect on glucocorticoids {β = −0.03, 89% CI = [−0.19, 0.16]}. In other words, when drought risk was held constant at 0 (seasonally appropriate rainfall), there was no difference in predicted glucocorticoid values for survivors and fatalities regardless of the season in which they were compared (Fig. 5). Moreover, survivors and fatalities both exhibited the expected seasonal increase in glucocorticoids during the dry season, as there was no strong evidence for an interaction between survival status and expected rainfall {β = 0.10, 89% CI = [−0.02, 0.22]; table S4}. It was in their response to drought risk during the wet season where they differed; as drought risk increased, survivors exhibited a larger increase in glucocorticoids compared to fatalities (Fig. 4).

## DISCUSSION

These results from wild capuchins provide, to our knowledge, the first evidence from primates that a more robust stress response is associated with an increase in survival (*12*). We took an individual reaction norm approach using universal but unpredictable stressors (i.e., droughts) to examine fitness outcomes for individuals across an El Niño catastrophe. The field of behavioral ecology is in need of formal evolutionary models for predicting how the stress response of subjects varies plastically with individual characteristics, such as developmental history, age, sex, season, current health, or experience (*32*). Such models need to additionally account for the unpredictability of the stressor (moving beyond predictable seasonal comparisons). Also, such models need to be developed using subjects in settings that select for appropriate stress responsivity. We still have a long way to go, but the first step is for more research programs to use an individual, reaction norm approach to stress research (*15*). It would be useful to see this approach replicated with other populations of capuchins and other types of stressors (e.g., social stressors, extreme floods, or extreme La Niña events) to know whether the patterns observed in this study are generalizable.

### Why do some capuchins appear to have a “better” response than others?

An adaptive HPA axis is characterized by low baseline glucocorticoids, a quick rise in response to a challenge, and a rapid return to baseline (*8*). It has been argued that primates—with their sophisticated cognitive abilities—may experience chronic psychosocial stress (*33*), which in humans can result in a desensitized or “dysregulated” HPA axis (*34*) that can present as chronically high baseline glucocorticoids, weak negative feedback mechanisms, and/or a weak glucocorticoid increase in response to challenges (*6*). Because the capuchins that died during El Niño (fatalities) did not show signs of a dysregulated HPA axis before their deaths (i.e., they had similar glucocorticoid levels to that of survivors when rainfall was seasonally appropriate, also exhibiting a positive response to annual decreases in rainfall as did survivors), why did they have a less-responsive HPA axis during times when it (presumably) was needed most? Much theory has focused on context-dependent evolutionary selection, where the same trait can have diametrically opposite fitness consequences in different settings (*33*). Natural selection may maintain individuals that differ in hormonal responses to challenges—each adaptive under specific circumstances, selecting for reaction norms with the highest relative fitness across all environments, the most common environments, or the most harsh environments (*35*).

Because of the direction of the natural experiment—an El Niño that produced unprecedented drought during the rainy season—our specific prediction was that survivors would show a more robust HPA response to drought during the wet season. For fatalities, we expected a lack of a response (a flat norm of reaction). However, this was not what we found. Fatalities appeared to demonstrate a negative slope, with increasing glucocorticoids in response to higher-than-expected rainfall. It is possible that this reversal reflects a biologically relevant pattern in which there are two physiological phenotypes: one that responds to higher-than-expected rainfall with an increase in cortisol and one that responds to lower-than-expected rainfall with an increase in cortisol. A catastrophically rainy few months at Barro Colorado Island, Panama, resulted in high mortality of white-faced capuchins (*36*). As periods of extended extreme rainfall are rare at Lomas Barbudal, we lack the natural experiment (e.g., a severe and prolonged La Niña event) to test this possibility. However, if these are two distinct phenotypes, it is possible that the individuals that survived the severe El Niño drought would not have survived a severe La Niña flood. A post hoc examination of capuchin foraging patterns in this population (fig. S4) shows a complex relationship between seasonal change and drought risk. Namely, higher drought risk was associated with reduced foraging for fruits in the early wet season months, but lower drought risk was associated with reduced foraging for fruits at the end of the wet season. Therefore, although “drought” and “flood” may both be associated with a reduction in food resources, these catastrophic events may select for different HPA axis phenotypes.

In addition, hormones can have pleiotropic effects, where hormone secretion may elevate the expression of one trait while decreasing the expression of another (*37*). Pleiotropic effects may make it impossible to achieve perfectly adapted HPA responsiveness when faced with a highly variable environment, filled with non–life-threatening, but nevertheless challenging, social stressors. Particularly when considering the “wear-and-tear” aspects of the HPA axis, frequent robust responses may carry different evolutionary consequences, such as a shorter lifespan (*11*).

Last, developmental experiences can alter lifelong stress responsivity in adaptive or nonadaptive ways (*38*, *39*). Developmental constraints have been shown to have lifelong consequences in primates (*40*). Prolonged exposure to stress early in life is associated with alterations in the HPA axis [via changes in density of corticotropin-releasing hormone binding sites and glucocorticoid receptors (*41*, *42*)], resulting in diminished negative feedback within the stress response system that persists into adulthood (*43*). Individuals that experienced greater earlylife adversity may be more likely to exhibit higher baseline- and response- glucocorticoids as adults because of an inability to turn off this physiological response.

### El Niño as a natural experiment

El Niño droughts can be catastrophic to natural populations. However, because we can anticipate these droughts with some accuracy, these events can provide rare natural experiments to help us understand some aspects of the adaptive HPA axis response to challenges. We should take advantage of these opportunities. One limitation to our study was that we had insufficient hormone samples during the El Niño event itself and therefore had to rely on previous stressors to gauge HPA responsivity for each individual. Because most of our most informative datasets on the relationship between the HPA axis and fitness can derive from natural disasters such as El Niño (*44*–*47*), we advocate that long-term field biology research continually seeks out early signals for natural disasters (like droughts or floods), remain “at the ready” for pivoting data collection when disasters strike, and monitors health and fitness across the event. Climate model predictions anticipate that global warming will exacerbate the frequency of natural disasters worldwide (*48*, *49*). While we cannot buffer our study populations from these events, we can use them to learn which aspects of the HPA axis help individuals to “weather the storm.”

## MATERIALS AND METHODS

### Study site and subjects

Data for these analyses come from the Lomas Barbudal Monkey Project, which was founded by S.E.P. in 1990 (*50*). The capuchins primarily range in the Reserva Biológica Lomas Barbudal (a government-protected nature reserve) and Hacienda Pelón de la Bajura (a private farm/ranch). This is a tropical dry forest habitat (*51*), consisting primarily of secondary deciduous forest, with some patches of older growth. There are also some patches of oak forest and savanna and many forest fragments in the northwestern and eastern edges of the study area because of farming and cattle grazing. The area is quite vulnerable to anthropogenic fire, one of the primary conservation challenges in the region (*52*).

All study subjects were from a habituated population of white-faced capuchins studied by Perry and colleagues since 1990 (*50*), with life histories from known individuals across 13 groups, 8 of which provided subjects for these analyses. In total, 19 adult female capuchins (using a conservative criterion of being 7 years old by the time of their death) died during the El Niño drought (15 May 2014 to 14 May 2016). All female disappearances were recorded as deaths because, with a few exceptions, female white-faced capuchins remain in their natal groups throughout their lives (*53*, *54*). The observed increase in female mortality during the El Niño drought (Fig. 1), as well as the difficulty in determining male deaths because of possible emigration in this female-philopatric species (*54*), led us to focus on females for this study.

#### Subject exclusion criteria

Although the project has amassed thousands of fecal samples from individuals in this population, at the time we selected samples to include in our analyses, none of the samples in this study had been analyzed previously for glucocorticoids (*21*). Rather than assaying all samples indiscriminately (and to reduce financial costs), we selected all females that died across El Niño (“fatalities”) and matched them with other females from the same social group that lived (“survivors”). Of the 19 fatalities, we excluded 5 females that did not have hormone samples from both wet and dry seasons or had fewer than 10 samples in total. Once subjects had been selected and lab work began, we found that some of the samples selected had insufficient mass to measure glucocorticoids; this brought the total number of fecal samples below 10 for three subjects (one fatality and two survivors), but we did not eliminate more subjects at this point. Thus, our sample size for this analysis was 28 females (14 fatalities and 14 survivors). Because capuchin groups are small, there were only two to six adult (>6 years old) females in each group for whom we had fecal samples. Of those, we aimed to select the female nearest in age and dominance rank who also had an adequate number of samples across both wet and dry seasons. The best match for rank was not always the best match for age. Sometimes, the same survivor female was the best match for more than one fatality, so we had to select the second best female for one of them. The rank at the time of the start of the drought averaged 0.47 (SD = 0.15) for fatalities and 0.52 (SD = 0.23) for survivors (1.0 being the highest ranking). The average age at the start of the drought was 16.1 years (SD = 7.9) for fatalities and 12.3 years (SD = 4.8) for survivors. We control for the confounding of age and rank in our models predicting survival.

### Female mortality

We determined mortality rate by using the number of female deaths and the total number of females available (“exposure”) in the study population each year. Across the 2-year El Niño drought, the average mortality rate was 17% compared to an average of 4% across all other years (Fig. 1). Although the Multivariate El Niño Southern Oscillation (ENSO) Index (MEI2) values do not strictly indicate that 2014 was an El Niño year, most of 2014 is classified with the 2015–2016 El Niño because it is characterized by severe drought (*18*). We ran a generalized linear model with a binomial distribution in R [version 4.3.0 (*55*)] to predict female mortality rates in each year since the long-term Lomas project began in 1990. We predicted rates on the basis of whether or not an El Niño drought occurred in that year, and we further distinguished the two most severe El Niño droughts since the Lomas study began (1997 and 2014 to 2016) (*18*). For this analysis, the year begins on May 15 and ends on May 14. The exposure (approximate number of adult females available) changes during the year, as females mature to adulthood or die during the year; in Fig. 1, this number is rounded to the nearest integer. Even when a drought extended to multiple years, each year was treated as a separate data point.

### Precipitation data

Daily rainfall has been recorded at the Lomas Barbudal site since June 2013 via a Hobo (R) rain gauge data logger (Onset, Cape Cod, MA), which is maintained by the Organization for Tropical Studies. Unfortunately, to match up with the hormone samples used in this study, we needed rainfall data from 2008 to 2013. Therefore, we used rainfall data from Palo Verde National Park, a tropical dry forest located 18.26 km south of Lomas, where daily precipitation has been recorded since September 1996. We calculated a 30-day rolling sum of rainfall at each site, and during years when data were available for both sites (July 2013 to June 2018), values were strongly correlated (ρ = 0.92, fig. S5).

#### Palo Verde dataset

The Palo Verde dataset had reference data available for September 1996 to December 2023. Specifically, we used 18 years of daily rainfall (1996 to 2013) from this dataset to calculate a precipitation index. The Palo Verde dataset was missing daily rainfall records for only 3% of days during the reference period, but no data were missing from the study period (2008 to 2013) or during the El Niño drought. We filled in missing data with the average daily rainfall for the 30 days surrounding the missing date, as long as at least half of the surrounding dates were not also missing rainfall data.

#### Precipitation and drought risk index

Our objective in computing a precipitation index was to quantify (for any given day) how dry the past month was, in comparison to what would be expected on the basis of a reference sample from other years. We used a metric (*56*) that consisted of the number of standard deviations from the mean, which we refer to as the precipitation index. We compared the 30-day total (or, equivalently, the 30-day mean) rainfall on a given day to corresponding data for the same month and day of the month in the reference years. In line with other research from tropical dry forests, we expected this 30-day window to correspond to important food resources for capuchins, including insect abundance (*57*) and plant phenology—particularly when the dry season switches to the wet season, a point when droughts have the most devastating effects (*18*). To evaluate typical rainfall for that time of year, we used the Palo Verde dataset described above as our reference dataset.

Drought is both challenging to define and monitor; as a result, using multiple indicators and indices for determining drought conditions has become a more common practice (*58*). We therefore use an alternative indicator (precipitation estimate source) and drought index to replicate our findings. This approach ensures that our results are replicable with a wider range of methodological approaches. Please see the Supplementary Materials and Methods for further descriptions of the alternate indicator and index.

#### Converting to a drought risk index

To make our models and all subsequent visualization more intuitive, we inverted our precipitation index so that positive values (which previously indicated higher-than-expected rainfall) indicated a higher risk of drought or “drought risk index.”

#### Expected rainfall

Our drought risk indices indicated when drier-than-expected conditions were present. However, we were primarily interested in the effect of drought during times of the year when there was a high expectation of rainfall (i.e., during the wet season). We therefore used our 30-day rolling mean rainfall estimates and standardized their values (i.e., created *z*-scores for rainfall). We used the mean *z*-score for each day to create a standardized rainfall estimate across the year, which captured seasonality in rainfall (fig. S6). We created this measure using only years with complete information before the start of the severe El Niño drought (i.e., 1997 to 2013). Values of 0 reflect average rainfall across the year, while values close to −1 capture the prolonged dry season, and values close to +1 capture times of the year with an expectation of heavy rainfall (i.e., the wet season).

### Demographic data

#### Female ages

Female ages were known or estimated. Most females (*n* = 18) had accurate birth dates because they were first seen as infants, and we could estimate their ages to the month (*n* = 14) or at least within the year (*n* = 4). Larger errors were assumed for females first seen as young juveniles (±1 to 2 years, *n* = 4). The remaining females (*n* = 6) were already parous when they were first observed, and their age estimates therefore have more uncertainty (±2 to 6 years). Age estimates for these females were based on physical appearances and ages of known offspring.

#### Group size and age at mortality

Groups were censused at least once per month whenever possible. In the few cases where the daily census was less reliable (i.e., group size greater than two individuals below max group size for that month and census takers spending <6 h conducting census because of poor viewing conditions), we used the median value of group size for that calendar month in that group. If a female disappeared, it was assumed that she died, because females are philopatric in this species. Only one case of female transfer to a different group has been observed, and this was an unusual circumstance (all her female kin had died, and she followed her dispersing son to a neighboring group). When females disappeared during an observation gap (i.e., between censuses), we assigned her death to the midpoint of the gap. We excluded one female whose window of possible death dates included some time before the El Niño drought started.

#### Reproductive state

We determined the reproductive state post hoc for each subject. Using the average gestation length of white-faced capuchins [~158 days (*59*)], females were categorized into “early pregnancy” (i.e., first half) or “late pregnancy” (i.e., second half) on the basis of estimated parturition date. Females were coded as nursing for 1 year past the birth of an infant unless the infant died during its first year of life, in which case they were coded as nursing for the period between birth and death of the infant. The average age at first birth is 6 years in Lomas capuchins (*60*), so females were coded as “pre-reproductive” if they were (i) younger than 5 years at sample collection and (ii) not coded as pregnant or nursing. All other situations were scored as “nonpregnant/non-nursing.”

### Hormone analysis and sample inclusion criteria

#### Hormone sample collection

Sample collection was opportunistic and required a positive identification of the individual at the moment of defecation. We collected fecal matter in latex gloves immediately following defecation between 5:00 and 18:00. Samples were not collected if they were contaminated with urine, fell into the river, or could not be differentiated from nearby defecations. Samples were kept on ice in thermoses until the return to camp (by 19:00). Samples were then frozen at −18°C until processing. Samples were dried in an oven at 95°C and then ground with a mortar and pestle, and undigested items and debris were removed using tweezers and/or a mesh tea strainer. Processed samples were stored out of direct sunlight in Whirl-Pak bags at ambient temperature (~27°C) for at most 8 months in Costa Rica before shipment. Following arrival at the University of Michigan, samples were stored at −20°C until extraction and analysis.

#### Hormone sample analysis

We assayed available samples from each female (*n* = 28 total females) on the basis of the following criteria. Samples were excluded if they (i) were collected outside of the study period (January 2008 to December 2013) or (ii) had <0.1 g of dry fecal matter available to extract. To avoid seasonal sampling bias, we did not use more than four samples from a female collected in the same month. If more than four samples were available for any month, we selected the four samples that were the most spread out according to collection date, and we never used multiple samples from the same day. For the days where more than one sample was available for a particular female, we selected the sample collected at the earliest time for consistency. Last, we did not select samples from the same individual collected on sequential days (e.g., if we had samples from 3 days in a row, we would select the first and last ones to assay). Of the 763 total samples, we removed 5 outliers [4 because glucocorticoid concentrations were more than 10 SD above the mean and may have been contaminated with urine and 1 because duplicate runs did not produce a coefficient of variation (CV) under 20%], leaving a final sample size of 758 samples (99.3%) from our 28 female subjects that contributed to analyses (mean of 27 ± 16 samples per female; range: 5 to 61 samples per female).

Before extraction, samples were brought to room temperature. From each sample, 0.15 g (minimum of 0.10 g) of dry fecal powder was added to a labeled tube (15-ml Falcon polypropylene tube) and then extracted with 2 ml of 80% EtOH. Further details of the extraction procedure can be found in (*21*). The supernatant at the end of the extraction was transferred to a labeled microcentrifuge tube with an O-ring cap and stored at −20°C until analysis.

All fecal samples were assayed for glucocorticoids using the DetectX Cortisol immunoassay kit from Arbor Assays (Arbor Assays, K003), which has been validated for use in the Lomas capuchin population (*21*). Briefly, samples were diluted with assay buffer (range: 1:5 to 1:180) and then added to the plate in duplicate (50 μl per well). After following the protocol from the manufacturer, plates were read using the Synergy HTX microtiter plate reader (BioTek, Santa Clara, CA) at a wavelength of 450 nm. Final concentrations were calculated as nanograms per gram using MyAssays software and accounting for sample dilution and dry fecal weight. High concentration and low concentration pools were run in all plates (*n* = 75 plates), and interassay CVs were 11.9 and 19.4%, respectively. Last, average intra-assay CVs were 8.4% (high pool) and 16.0% (low pool), indicating the absence of drift.

### Data analyses

We conducted four primary analyses. The first one was a preliminary analysis to identify which factors affected glucocorticoid levels (factors that would then need to be controlled for in our subsequent analyses). We constructed a Bayesian LMM with glucocorticoid metabolites (log-transformed) as the outcome variable. As predictor variables, we focused on general factors known to affect glucocorticoids in wild primates: collection time (in minutes, continuous), age (continuous), rank (continuous), group size (continuous), and reproductive state (categorical). Continuous control variables were scaled to a mean of zero and a standard deviation of one. We also included expected rainfall (continuous) for a given date. Individual ID and group ID were included as random effects. As expected, model results indicated that glucocorticoids were higher in samples collected during (a) drier periods (when expected rainfall was low), (b) progressive stages of pregnancy, and (c) earlier times of the day (table S2). As found in another wild capuchin population (*59*), glucocorticoid concentrations did not differ between pre-reproductive, nursing, and nonpregnant/non-nursing females (table S2). We therefore grouped these categories together as “not pregnant” to compare against the “early pregnancy” and “late pregnancy” for our primary analyses.

Second, to examine how reaction norms predicted survival, we needed to obtain reaction norms for each subject (reaction-norm-extraction model). To do this, we constructed a Bayesian LMM, with glucocorticoids (log-transformed) as our outcome variable, examining the effects of expected rainfall (continuous), drought risk index (reverse-coded precipitation index, continuous), and their interaction. Because sample collection time and reproductive state were found to predict glucocorticoids in the preliminary model above (table S2), we controlled for these two variables. We also included individual ID and group ID as random effects. Furthermore, we included random slopes by individual ID for all fixed effects (including the interaction between the drought risk index and the expected rainfall), allowing us to examine individual plasticity in stress responsivity to drought (*15*). We extracted BLUPs for each individual’s intercepts and slopes against our drought indices (i.e., individual-level reaction norms to drought risk). Specifically, we extracted values for intercept and slope at expected rainfall of 1, which corresponded to the wet season (fig. S6).

Our subsequent third and fourth analyses comprised testing whether glucocorticoid reaction norms predicted survival (glucocorticoids-predicting-survival model; see main text for details) and testing whether survival predicted glucocorticoids (survival-predicting-glucocorticoids model; see main text for details).

All statistical models were fit under a Bayesian framework using the “brms” package [version 2.19.0 in R (*61*)]. We used four Markov chains with 4000 iterations each, including a warmup of 1000 iterations and a thinning of 1. For Gaussian models, we used default (flat) priors for fixed effects and default weakly informative half-Student-*t* priors for the random effects. We assessed chain convergence with $\hat{R}$ values of 1.01 or less, effective sample size of at least 600, and visual inspection of trace plots for each model’s parameters (tables S1 and S3 to S5 and figs. S7 to S9). We assessed model fit graphically with posterior predictive checks via the pp_check() function in “brms.”

### Ethics statement

This research protocol was approved by University of Michigan IACUC (protocol no. 3081), University of California, Los Angeles’s Animal Research Committee (ARC nos. 1996-122 and 2005-084 and various renewals) and University of California, Los Angeles’s Institutional Biosafety Committee (211.10.0-r and various renewals) and was performed in accordance with the laws of Costa Rica and the ethical principles of the American Society of Primatology.

## Supplementary Materials

### This PDF file includes:

Supplementary Materials and Methods Figs. S1 to S9 Tables S1 to S5 References
