# Supplementary material for: Stress responsiveness in a wild primate predicts survival across an extreme El Niño drought
Source: Sci Adv. 2025 Jan 22;11(4):eadq5020. doi: 10.1126/sciadv.adq5020 (PMC11753399; doi:10.1126/sciadv.adq5020)
Supplement: Supplementary file 1 — Supplementary Materials and Methods Figs. S1 to S9 Tables S1 to S5 References [file sciadv.adq5020_sm.pdf]

Science Advances
AAAS

Supplementary Materials for

Stress responsiveness in a wild primate predicts survival across an extreme El Niño drought

Sofia C. Carrera *et al.*

Corresponding author: Susan E. Perry, sperry@anthro.ucla.edu

Sci. Adv. **11**, eadq5020 (2025)
DOI: 10.1126/sciadv.adq5020

This PDF file includes:

Supplementary Materials and Methods
Figs. S1 to S9
Tables S1 to S5
References

SUPPLEMENTARY MATERIALS AND METHODS

Alternative methods for measuring drought

We replicated our analyses using a modification of a commonly used drought index (the Standardized Precipitation Index, or SPI (*56*), (*62*), as well as an alternative dataset, CHELSA (*63*, *64*), **(see fig. S1**). Results from our replicated analyses are presented in the SI in **tables S2,S3 and S5** and **fig. S2** and **fig. S3**. In all, four analyses were run using both drought indices (the drought index described in the main text and SPI), each calculated with both sets of climate data (Palo Verde and CHELSA).

CHELSA data set

CHELSA (Climatologies at High Resolution for the Earth's Land Surface Areas, version 2.1, https://chelsa-climate.org), (*63*,*64*)) provides high resolution global estimates of surface precipitation based on cloud cover-informed downscaling. We downloaded daily precipitation estimates from CHELSA for 1980-2016 and extracted values for the area of our study site.

Precipitation and drought risk indices

The SPI (Standard Precipitation Index) is a widely used measure of rainfall in the literature to quantify droughts (*62*). We initially used the 'standaRdized' package in R (*65*) to compute SPI, but (like others (*66*)), we encountered problems stemming from the high frequency of zero rainfall values in the dry season, which led to implausible results (e.g., zero rainfall being assigned a positive value, when zero cannot logically be above average). The first problem was that the program returned 'NA' in cases where zeros were too common in the reference data. This problem could be ameliorated by adjusting the 'standaRdized' parameter (ref.na.thres) in two ways: (1) If n out of m reference values were zero (where m is the number of reference years), we considered a zero value to be at percentile $n/2m$ rather than n/m (e.g., if 7 of 10 reference values were zero, we viewed zero as the 35th percentile (dryer than normal), not the 70th (wetter than normal)). (2) The other change kept the estimates within ranges plausibly supported by the data (e.g., if none of the reference data were zero, then a zero value would be interpreted by 'standaRdized' as minus infinity). Instead, we treated any value below the smallest reference value as $100/2m$'th percentile: e.g. if there were 10 reference years, we called anything below the minimum the 5th percentile. Similarly, anything above all 10 reference values would be called the 95th percentile.

The problems with the SPI suggested that the simpler precipitation index used in the main text would better serve our purposes. For example, if the mean and standard deviation for Jan 30, 2015 in the reference years were 10mm and 5mm, then a value of 13mm on Jan 30 would be assigned the value of (13-10)/5=0.6) using the precipitation index approach from the main text. In theory, this precipitation index in the main text could produce NA or implausible values (e.g., if all of the reference data were zero), but we did not observe such problems in our data.

Converting to a drought risk index

As in the main text, for all models and all subsequent visualizations, we inverted our precipitation indices, so that positive values (which previously indicated higher than expected rainfall) indicated a higher risk of drought, or "drought risk index".

Expected rainfall

As was done in the main text for the Palo Verde data set, we created measures of expected rainfall for the CHELSA data set, using only years with complete information prior to the start of the severe El Niño drought.

SUPPLEMENTARY FIGURES AND TABLES

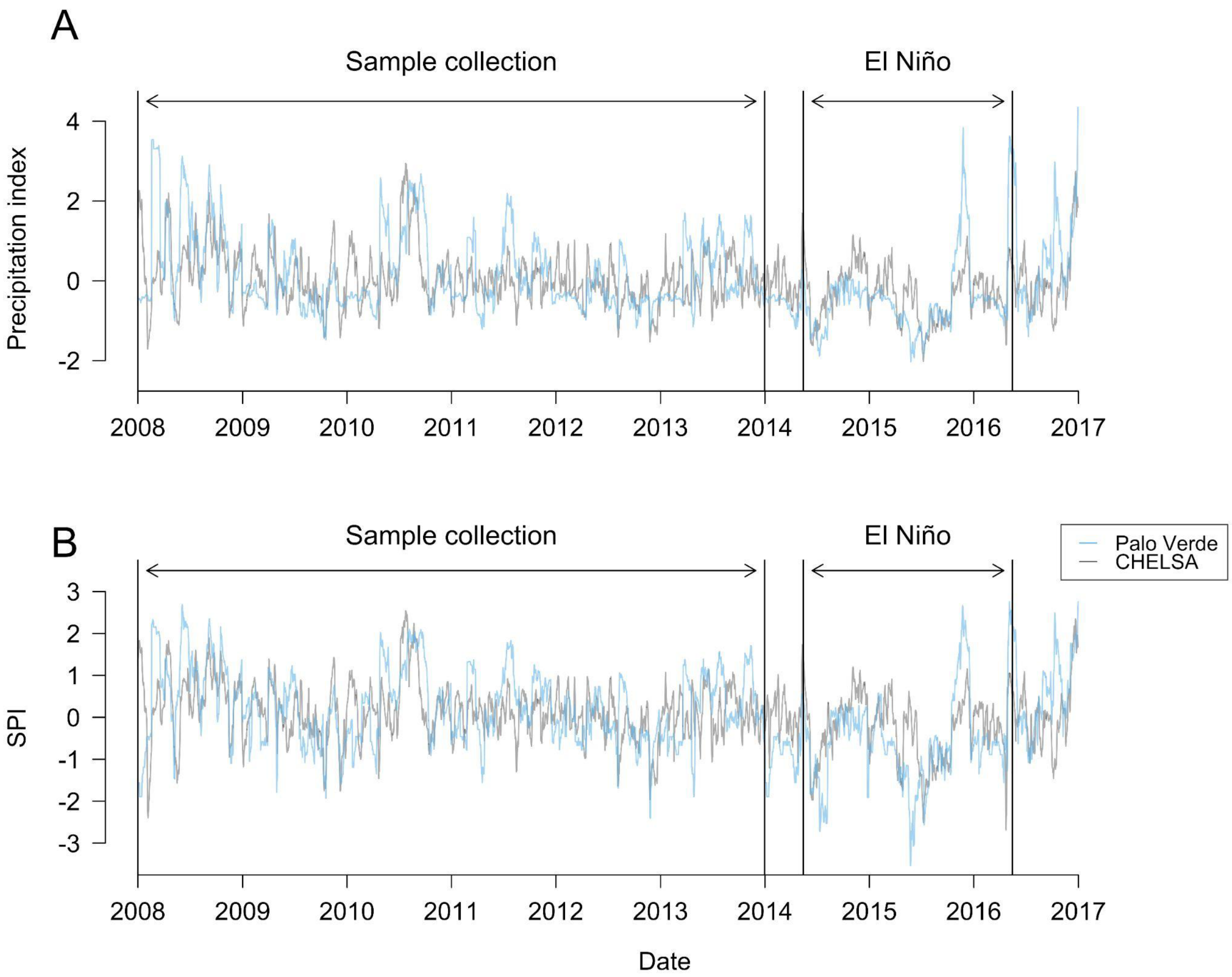

Fig. S1. Precipitation indices capture the severity of the 2014-2016 El Niño drought. All estimated rainfall measures used in primary and supplementary analyses for our period of interest (2008-2016). (A) Blue lines show the precipitation index (described in the main text) calculated over the past 30 days at Palo Verde, gray lines represent the same for CHELSA. (B) Blue lines show the SPI calculated over the past 30 days at Palo Verde, gray lines represent the same for CHELSA.

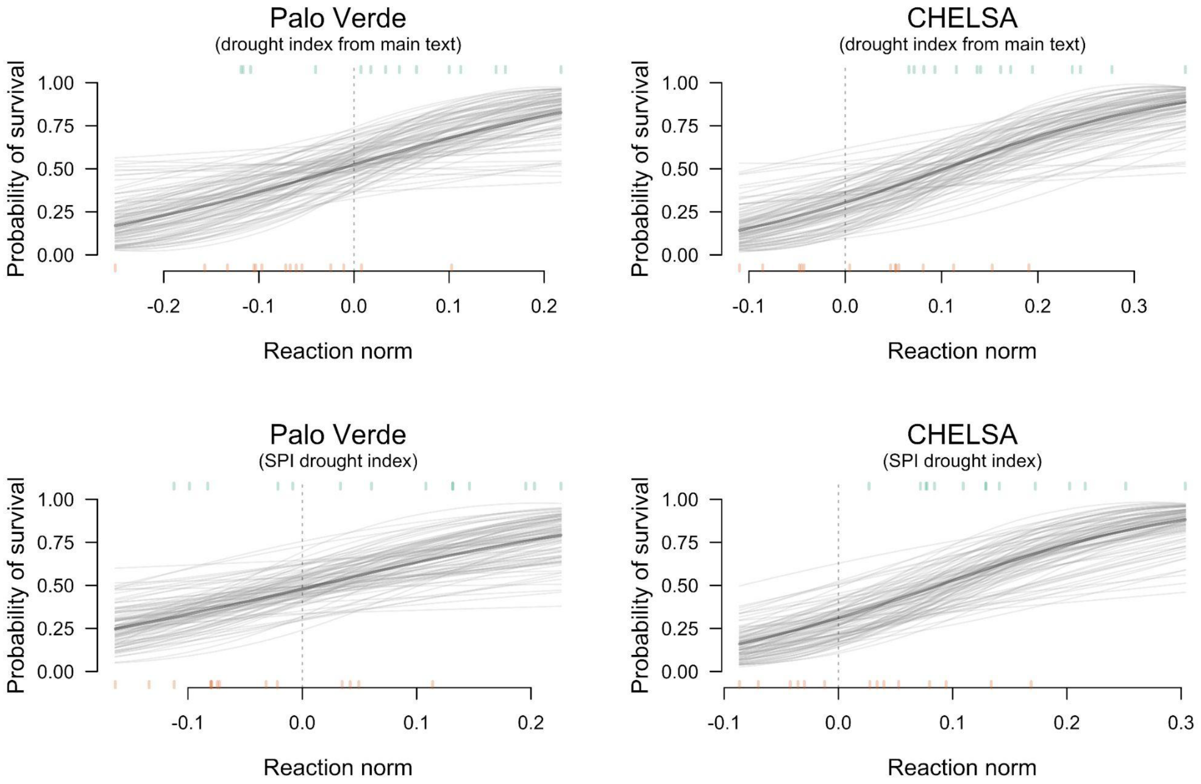

Fig. S2. Capuchins with an increase in glucocorticoids during wet-season droughts were more likely to survive El Niño. The probability of survival increased as the reaction norm (i.e., BLUP of slope) increased, or in other words, individuals were more likely to survive if they were predicted to have higher glucocorticoids with increasing drought risk index. These figures replicate **Fig. 3** (from main text) but verify results using two different drought indices (the one from the main text and SPI drought index) and two different climate datasets (Palo Verde and CHELSA). The thin gray lines represent 100 random draws from each posterior distribution and the thick black line represents the estimated population mean.

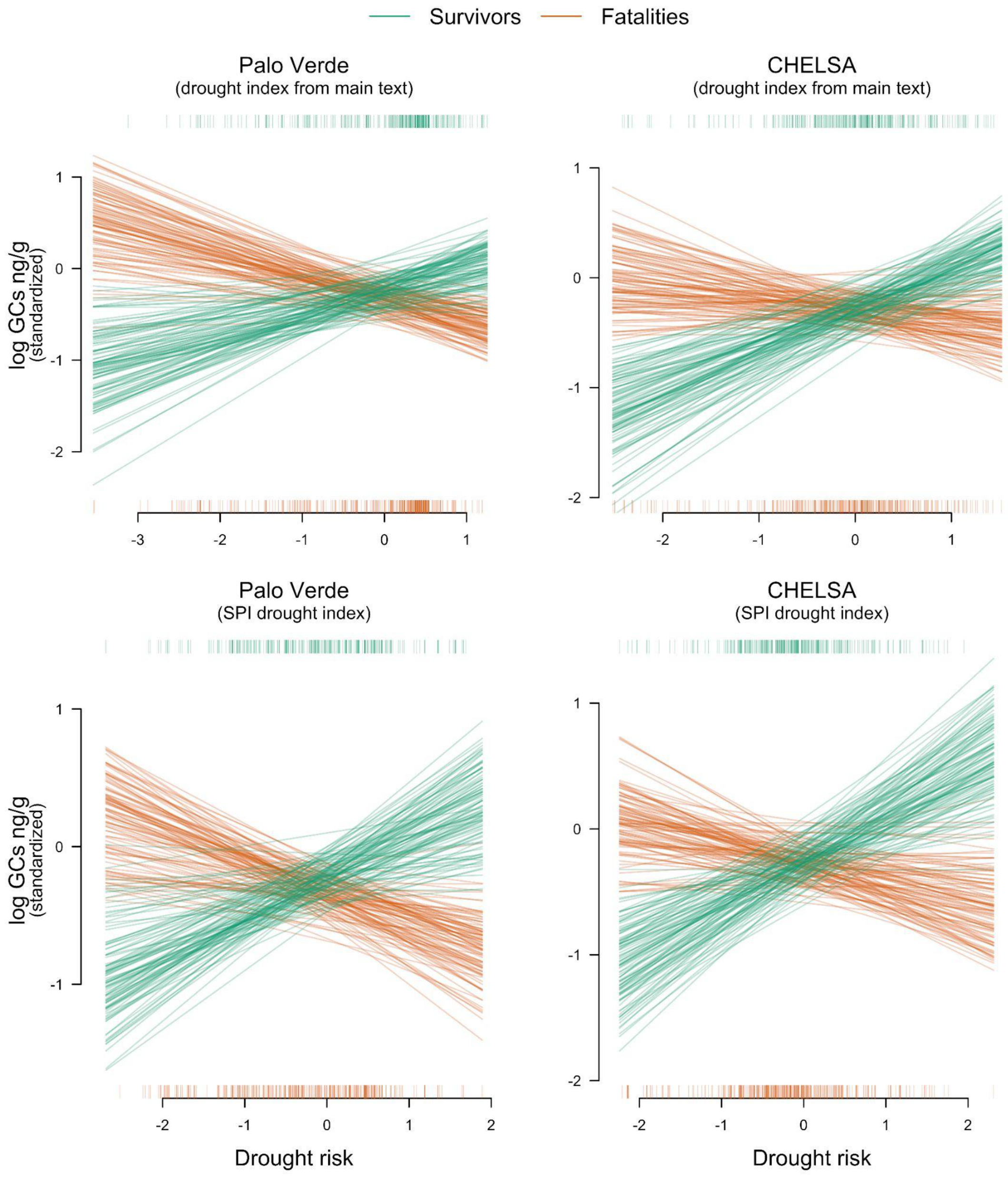

Fig. S3. Survivors of El Niño had higher glucocorticoids during previous droughts compared to fatalities. During the wet season when droughts can have a catastrophic effect on food supply for capuchins, individuals that survived the El Niño drought exhibited an increase in glucocorticoids from non-droughts to droughts. Green and orange lines represent 100 draws from the posterior distribution. These figures replicate **Fig. 4** (in main text) but verify results using two different drought indices (the one described in the main text and the SPI drought index) and two different climate datasets (Palo Verde and CHELSA). Expected rainfall was a continuous measure, but here we show the effect of drought risk at times of the year with higher expected rainfall (i.e., standardized expected rainfall values of 1, which corresponds to the wet season).

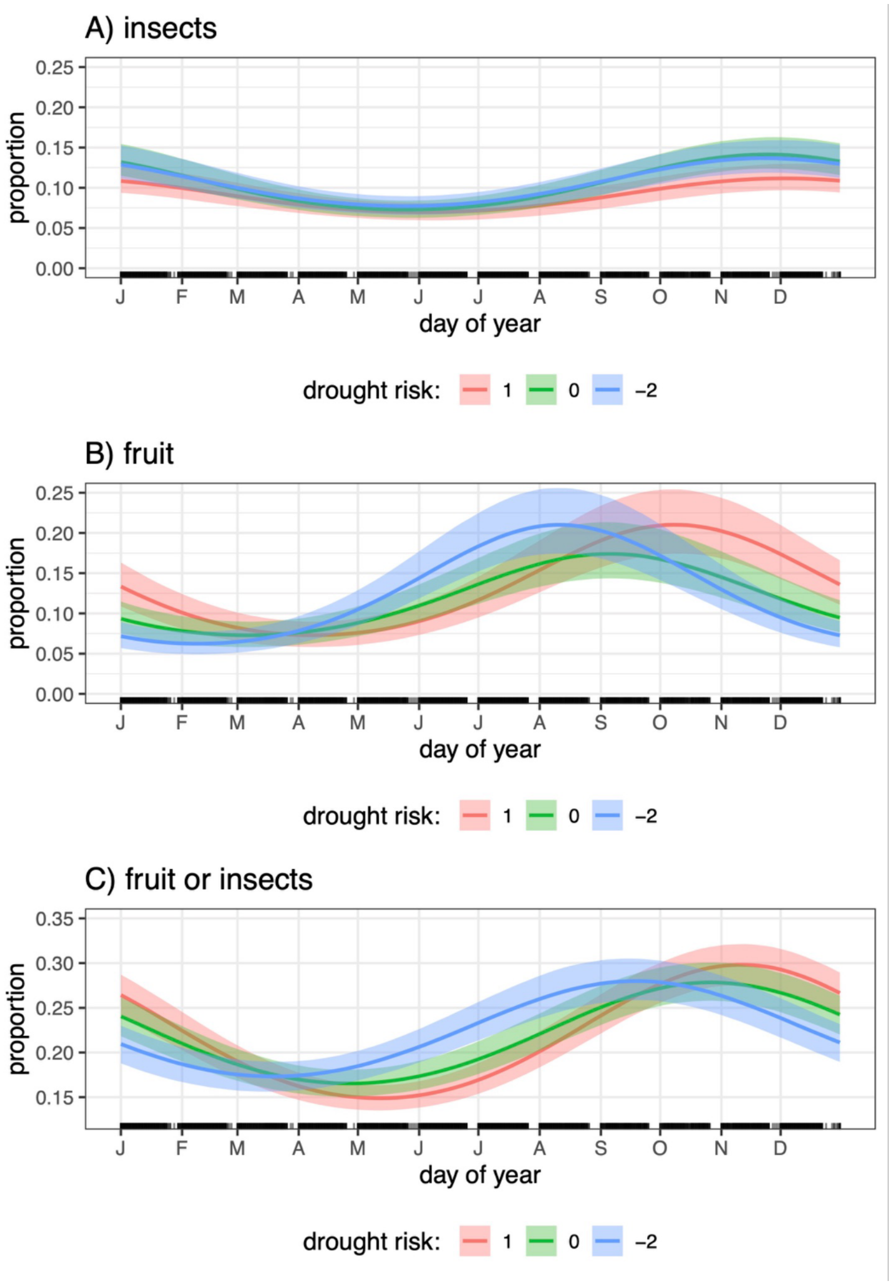

Fig. S4. Post-hoc analysis of capuchin foraging behavior. The figure shows the proportion of time that adult capuchins spent handling and/or eating their primary food sources of A) insects, B) fruit, and C) insects or fruit. Figures were generated using the *conditional_effects()* function from the 'brms' package, with the number of trials set to 1. Drought risk was a continuous variable in this model (See SI code for foraging models for additional details). Drought risk values during this period (2008-2013) varied from -3.54 to 1.48. Drought risk values of 1 (red), 0 (green), and -2 (blue) were chosen from within this range for illustrative purposes to represent dry, typical, and heavy rain conditions respectively. The rains typically begin around mid-May and end in mid-November (see fig. S6).

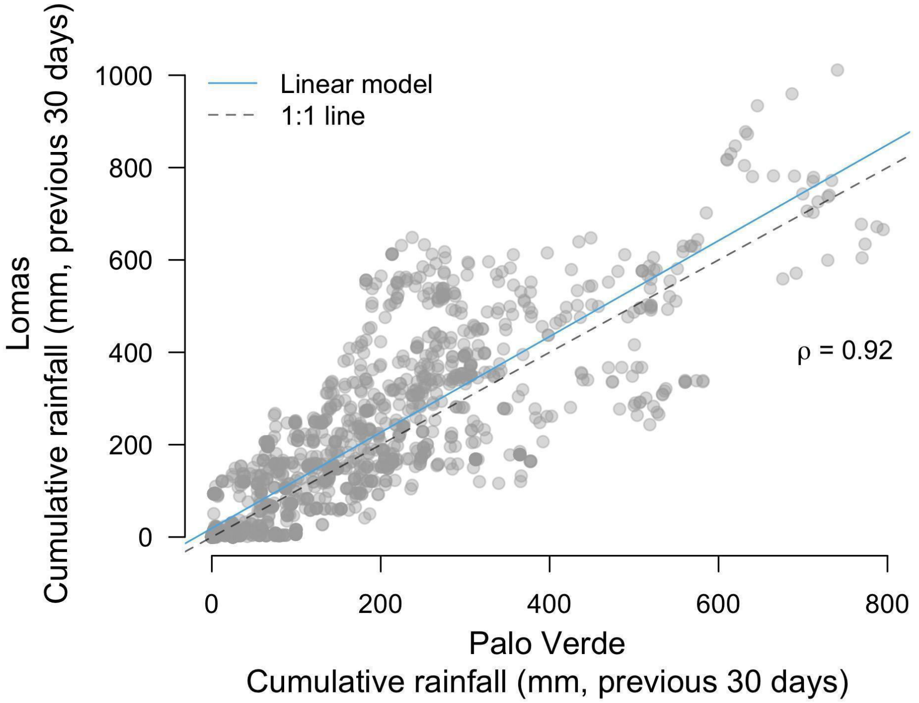

Fig. S5. Cumulative rainfall values are highly correlated between the Lomas Barbudal field site and the nearby Palo Verde station. Rainfall data for this study came from Palo Verde, a research site 18 km south of Lomas. Overlapping rainfall collection (Jul 2013-Jun 2018) revealed that cumulative rainfall (30-day rolling sums) at these sites were highly correlated ($\rho = 0.92$). Blue line represents simple linear regression. Dotted black line represents the 1:1 line.

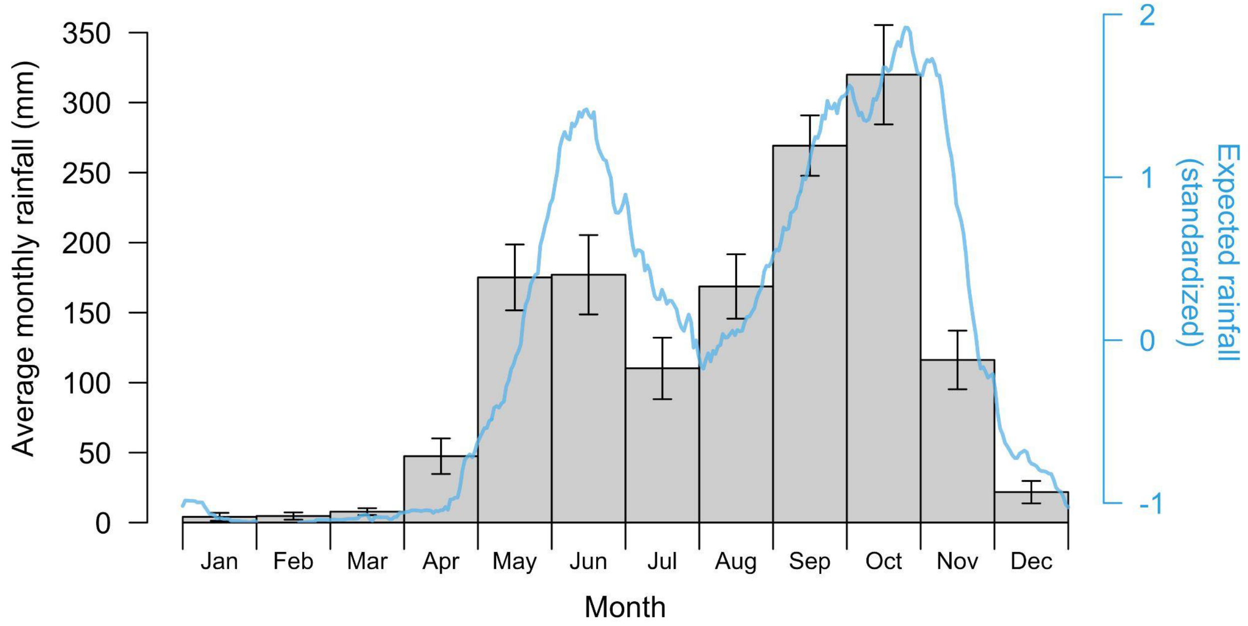

Fig. S6. Rainfall is highly seasonal at the Lomas Barbudal field site. Barplot of the average total rainfall (± SE) in each month at Palo Verde (Jun 1996 - Dec 2016). The blue line reflects the expected rainfall measure (z-score) used in our primary analyses, which captures the seasonal pattern of expected rainfall across the year.

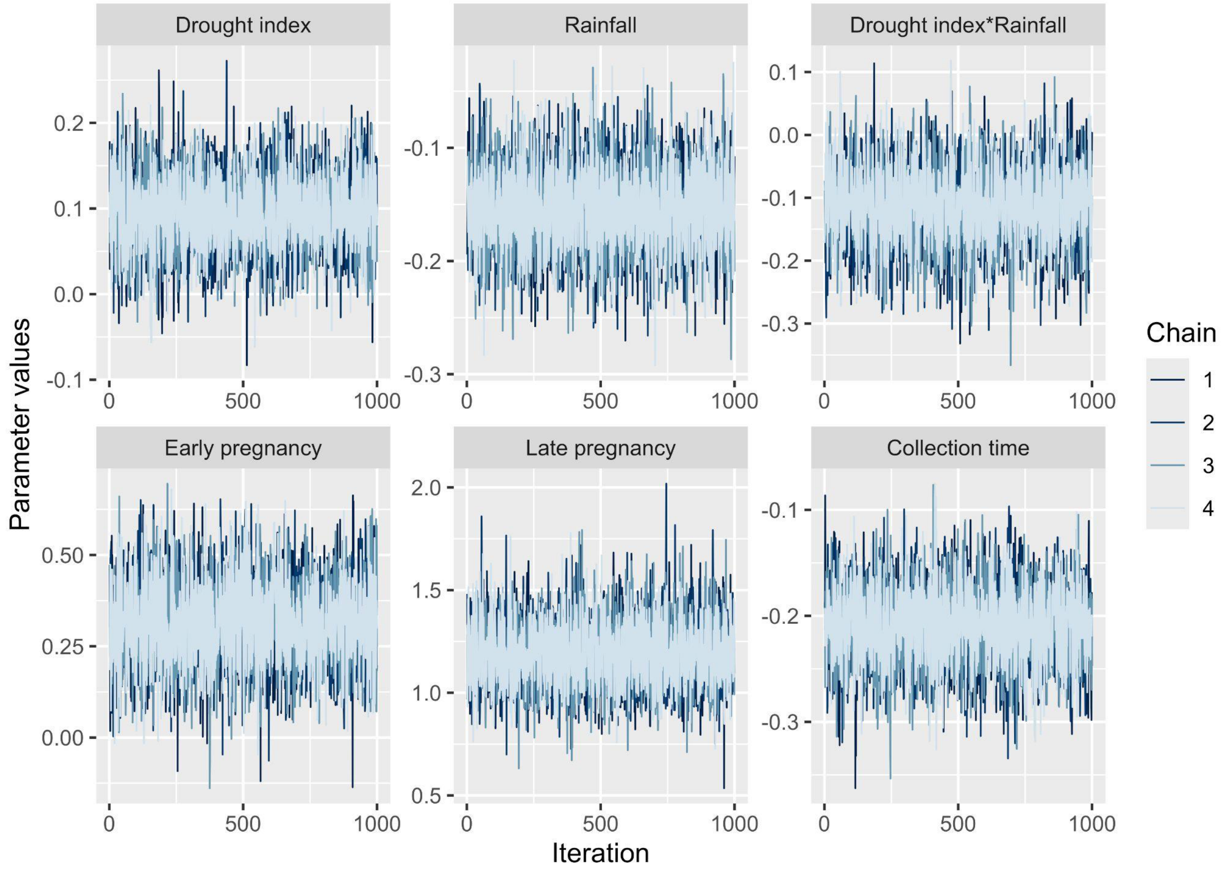

Fig. S7. MCMC diagnostic trace plots of parameter estimates in the *Reaction-Norm-Extraction Model* based on the drought index from the main text and expected rainfall from the Palo Verde dataset. The four Markov chains showed good convergence to the same posterior distributions for each parameter. Note the different scales on the y-axes.

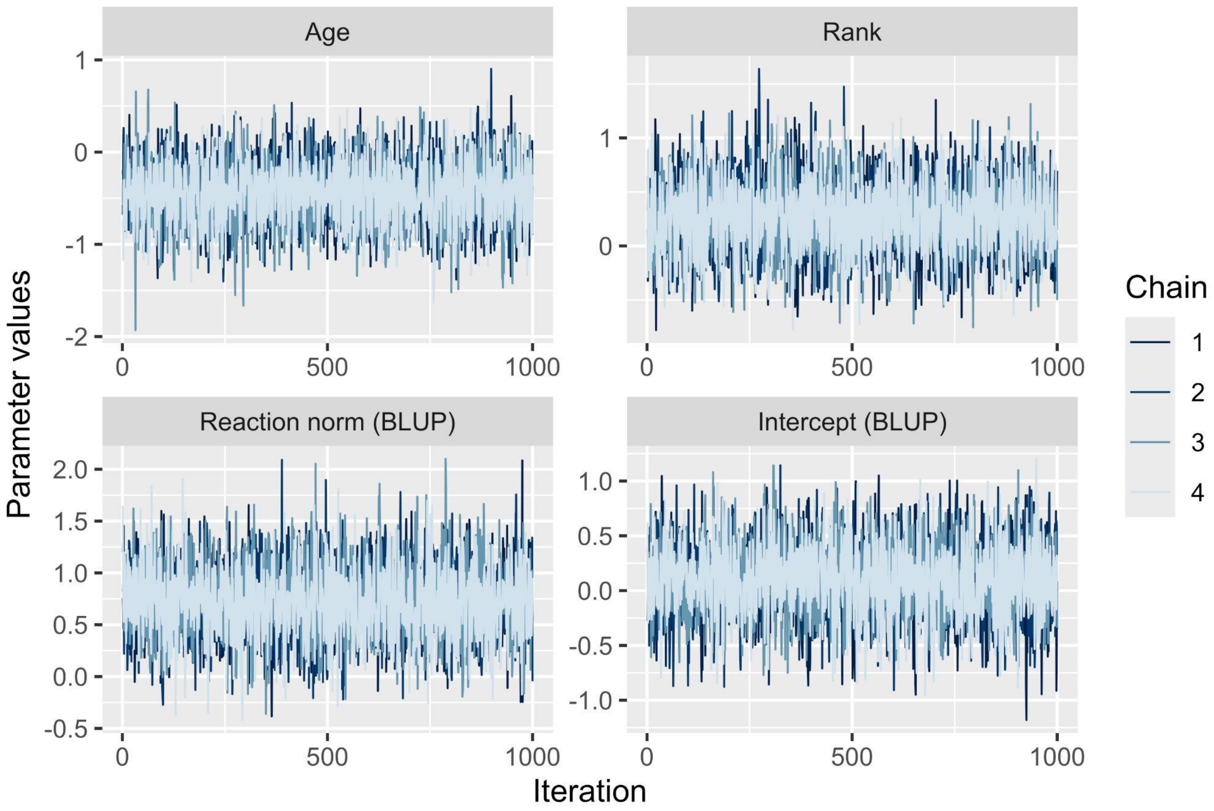

Fig. S8. MCMC diagnostic trace plots of parameter estimates in the *Glucocorticoids- Predicting-Survival Model* based on the drought index from the main text and the Palo Verde dataset. The four Markov chains showed good convergence to the same posterior distributions for each parameter. Note the different scales on the y-axes.

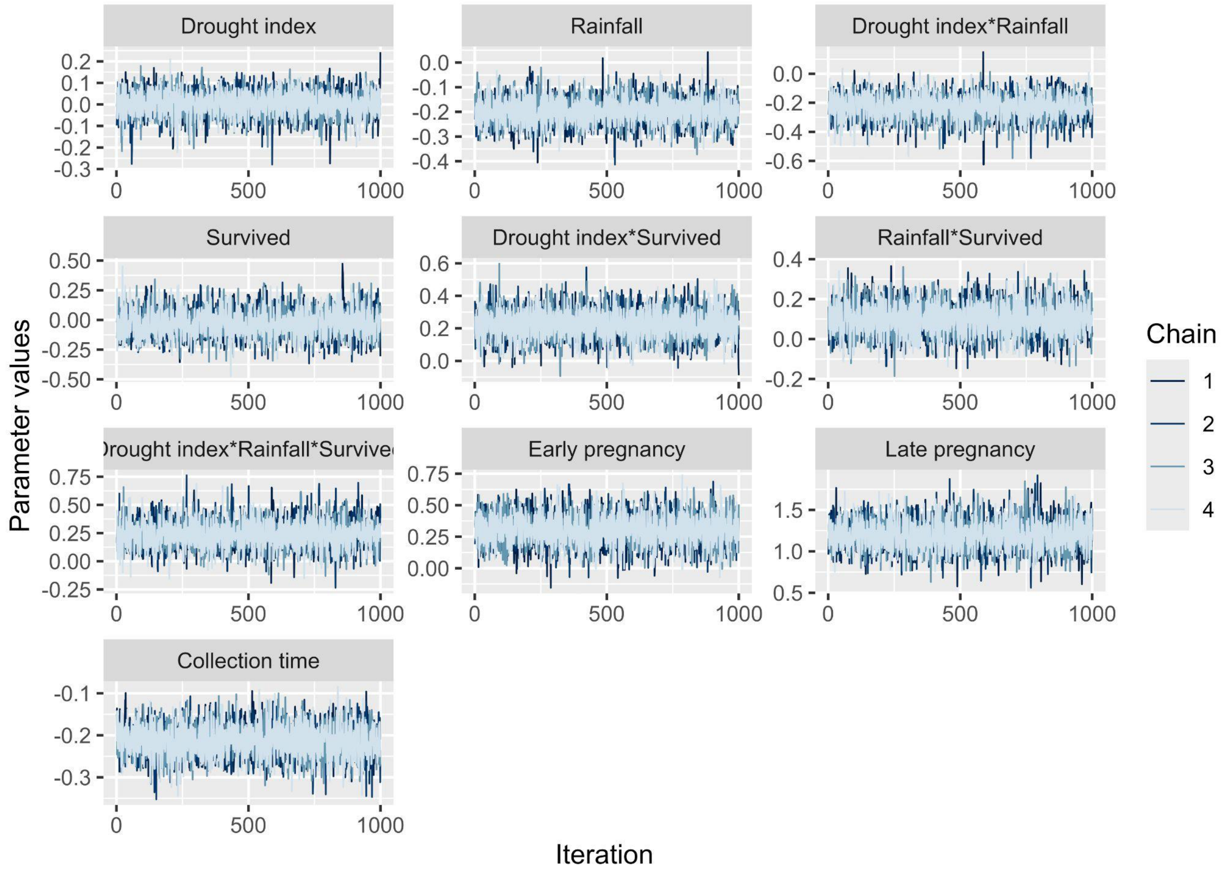

Fig. S9. MCMC diagnostic trace plots of parameter estimates in the *Survival-Predicting-Glucocorticoids Model* based on the drought index from the main text and expected rainfall from the Palo Verde dataset. The four Markov chains showed good convergence to the same posterior distributions for each parameter. Note the different scales on the y-axes.

	Parameter	Estimate	Est. Error	89% CI	Bulk ESS	Tail ESS	$\hat{R}$
**Population-level effects**							
	Drought risk	0.10	0.04	**[0.02, 0.17]**	4935	2794	1.00
	Expected rainfall	-0.15	0.04	**[-0.21, -0.10]**	7025	3533	1.00
	Drought risk * expected rainfall	-0.11	0.06	**[-0.21, -0.02]**	4106	3049	1.00
	Early pregnancy	0.31	0.12	**[0.13, 0.50]**	5224	3327	1.00
	Late pregnancy	1.19	0.16	**[0.95, 1.46]**	4839	3085	1.00
	Collection time	-0.21	0.04	**[-0.27, -0.15]**	5103	3053	1.00
**Group-level effects**							
ID (28 levels)	sd(Intercept)	0.19	0.08	[0.06, 0.32]	819	1048	1.01
	sd(Drought risk)	0.08	0.06	[0.01, 0.18]	1692	2267	1.00
	sd(Expected rainfall)	0.05	0.04	[0.00, 0.12]	2403	2578	1.00
	sd(Drought risk * expected rainfall)	0.18	0.08	[0.05, 0.31]	1419	1255	1.00
GroupID (8 levels)	sd(Intercept)	0.16	0.10	[0.04, 0.33]	1315	1061	1.00

Table S1. ***Reaction-Norm-Extraction Model* summary and diagnostics for the Bayesian LMM predicting log glucocorticoids used to generate norms of reaction (BLUPs).** Based on the drought index and Palo Verde dataset used in the model from the main text. The model also included random slopes for all fixed effects at the level of ID.

	Palo Verde		CHELSA	
**Parameter**	**Estimate**	**89% CI**	**Estimate**	**89% CI**
Expected rainfall	-0.18	**[-0.24, -0.11]**	-0.15	**[-0.21, -0.09]**
Early pregnancy	0.30	**[0.10, 0.49]**	0.30	**[0.09, 0.50]**
Late pregnancy	1.30	**[1.04, 1.59]**	1.28	**[1.02, 1.56]**
Nursing	0.05	[-0.12, 0.21]	0.04	[-0.12, 0.21]
Prereproductive	0.15	[-0.14, 0.45]	0.17	[-0.12, 0.46]
Age	0.07	[-0.05, 0.18]	0.07	[-0.04, 0.19]
Rank	-0.07	[-0.16, 0.03]	-0.07	[-0.17, 0.04]
Group size	0.03	[-0.08, 0.13]	0.03	[-0.07, 0.14]
Collection time	-0.21	**[-0.27, -0.15]**	-0.21	**[-0.27, -0.15]**
Observations	758		758	
Bayes R^2	0.292		0.285	

Table S2. Analysis of factors affecting fecal glucocorticoids. Model results for predictors of interest in the LMM testing effects of reproductive state, age, rank, group size, and expected rainfall on glucocorticoid concentrations. Samples collected from nursing and pre-reproductive females did not differ from those collected from cycling/other females, so these three categories were grouped together for further analyses as "not pregnant" (our reference category in later analyses). These models also included the random effects of ID and group ID, and random slopes for all fixed effects at the level of ID.

	Palo Verde				CHELSA			
	Drought index from main text		SPI		Drought index from main text		SPI	
**Parameter**	**Estimate**	**89% CI**	**Estimate**	**89% CI**	**Estimate**	**89% CI**	**Estimate**	**89% CI**
Reaction norm slope	0.73	**[0.17, 1.28]**	**0.70**	**[0.15, 1.27]**	**0.91**	**[0.33, 1.54]**	**0.90**	**[0.32, 1.50]**
Reaction norm intercept	0.09	[-0.44, 0.62]	0.13	[-0.41, 0.68]	0.09	[-0.45, 0.64]	0.09	[-0.46, 0.64]
Age	-0.44	[-0.98, 0.10]	-0.47	[-0.99, 0.06]	-0.42	[-0.96, 0.13]	-0.43	[-0.97, 0.09]
Rank	0.27	[-0.26, 0.80]	0.28	[-0.26, 0.83]	0.16	[-0.39, 0.72]	0.14	[-0.43, 0.71]
Observations	28		28		28		28	
Bayes R^2	0.194		0.189		0.243		0.235	

Table S3. Verification of *Glucocorticoids-Predicting-Survival Model* results (predicting survival from reaction norm BLUPs, with predictor variables scaled) using two different drought indices (the drought index from the main text, SPI drought index) and two different climate datasets (Palo Verde, CHELSA). Model output is on the logit scale.

	Parameter	Estimate	Est. Error	89% CI	Bulk ESS	Tail ESS	$\hat{R}$
**Population-level effects**							
	Drought risk	0.00	0.06	[-0.09, 0.09]	3841	2916	1.00
	Expected rainfall	-0.20	0.05	**[-0.28, -0.12]**	4770	3419	1.00
	Survivor	-0.03	0.11	[-0.19, 0.16]	3267	2785	1.00
	Drought risk * expected rainfall	-0.23	0.08	**[-0.36, -0.11]**	3377	2790	1.00
	Drought risk * survivor	0.23	0.09	**[0.09, 0.36]**	4491	3056	1.00
	Expected rainfall * survivor	0.10	0.08	[-0.02, 0.22]	4598	3293	1.00
	Drought risk * expected rainfall * survivor	0.24	0.12	**[0.06, 0.43]**	3674	2931	1.00
	Early pregnancy	0.31	0.12	**[0.12, 0.49]**	5335	3239	1.00
	Late pregnancy	1.19	0.17	**[0.94, 1.48]**	3707	2712	1.00
	Collection time	-0.21	0.04	**[-0.27, -0.15]**	4953	3326	1.00
**Group-level effects**							
ID (28 levels)	sd(Intercept)	0.17	0.08	[0.03, 0.30]	866	1192	1.00
	sd(Drought risk)	0.06	0.05	[0.01, 0.15]	2521	2901	1.00
	sd(Expected rainfall)	0.05	0.04	[0.00, 0.12]	2412	2535	1.00
	sd(Drought risk * expected rainfall)	0.14	0.08	[0.02, 0.28]	1500	1917	1.00
GroupID (8 levels)	sd(Intercept)	0.17	0.10	[0.04, 0.34]	1676	1420	1.00

Table S4. ***Survival-Predicting-Glucocorticoids Model*** **summary and diagnostics** for the Bayesian LMM (predicting glucocorticoids based on survival status). Based on the drought index from the main text and the Palo Verde dataset. The model also included random slopes for all fixed effects (other and Survival) at the level of ID.

	Palo Verde				CHELSA			
	Drought index from main text		SPI		Drought index from main text		SPI	
**Parameter**	**Estimate**	**89% CI**	**Estimate**	**89% CI**	**Estimate**	**89% CI**	**Estimate**	**89% CI**
Drought risk	0.00	[-0.09, 0.09]	-0.01	[-0.11, 0.09]	0.01	[-0.10, 0.12]	0.01	[-0.10, 0.12]
Expected rainfall	-0.20	**[-0.28, -0.12]**	-0.22	**[-0.30, -0.14]**	-0.17	**[-0.26, -0.08]**	-0.19	**[-0.27, -0.10]**
Survivor	-0.03	[-0.19, 0.16]	0.00	[-0.17, 0.16]	-0.06	[-0.22, 0.12]	-0.07	[-0.23, 0.11]
Drought risk * expected rainfall	-0.23	**[-0.36, -0.11]**	-0.22	**[-0.34, -0.10]**	-0.15	**[-0.29, 0.00]**	-0.15	**[-0.30, 0.00]**
Drought risk * survivor	0.23	**[0.09, 0.36]**	0.23	**[0.09, 0.37]**	0.07	[-0.09, 0.24]	0.08	[-0.08, 0.25]
Expected rainfall * survivor	0.10	[-0.02, 0.22]	0.13	**[0.01, 0.25]**	0.04	[-0.09, 0.16]	0.08	[-0.05, 0.20]
Drought risk * expected rainfall * survivor	0.24	**[0.06, 0.43]**	0.28	**[0.10, 0.46]**	0.45	**[0.23, 0.67]**	0.42	**[0.22, 0.64]**
Early pregnancy	0.31	**[0.12, 0.49]**	0.30	**[0.11, 0.50]**	0.31	**[0.12, 0.51]**	0.31	**[0.11, 0.49]**
Late pregnancy	1.19	**[0.94, 1.48]**	1.23	**[0.97, 1.51]**	1.24	**[0.97, 1.53]**	1.23	**[0.97, 1.53]**
Collection time	-0.21	**[-0.27, -0.15]**	-0.21	**[-0.27, -0.15]**	-0.20	**[-0.25, -0.14]**	-0.20	**[-0.26, -0.14]**
Observations	758		758		758		758	
Bayes R2	0.3		0.295		0.282		0.281	

Table S5. Verification of *Survival-Predicting-Glucocorticoids Model* results (predicting glucocorticoids based on survival status) using two different drought indices (drought index from the main text, SPI drought index) and two different climate datasets (Palo Verde, CHELSA). These models also included the random effects of ID and group ID, and random slopes for all fixed effects (other than Survival) at the level of ID.

REFERENCES AND NOTES

1. H. Selye, A syndrome produced by diverse nocuous agents. *Nature* **138**, 32 (1936).

2. B. N. Harris, Stress hypothesis overload: 131 hypotheses exploring the role of stress in tradeoffs, transitions, and health. *Gen. Comp. Endocrinol.* **288**, 113355 (2020).

3. J. Maniam, C. Antoniadis, M. J. Morris, Early-life stress, HPA axis adaptation, and mechanisms contributing to later health outcomes. *Front. Endocrinol.* **5**, 73 (2014).

4. T. E. Seeman, B. H. Singer, J. W. Rowe, R. I. Horwitz, B. McEwen, Price of adaptation–allostatic load and its health consequences. *Arch. Intern. Med.* **157**, 2259–2268 (1997).

5. A. Danese, B. S. McEwen, Adverse childhood experiences, allostasis, allostatic load, and age-related disease. *Physiol. Behav.* **106**, 29–39 (2012).

6. R. M. Sapolsky, L. M. Romero, A. U. Munck, How do glucocorticoids influence stress responses? Integrating permissive, suppressive, stimulatory, and preparative actions. *Endocr. Rev.* **21**, 55–89 (2000).

7. M. Kivimäki, A. Bartolomucci, I. Kawachi, The multiple roles of life stress in metabolic disorders. *Nat. Rev. Endocrinol.* **19**, 10–27 (2023).

8. C. W. Breuner, S. H. Patterson, T. P. Hahn, In search of relationships between the acute adrenocortical response and fitness. *Gen. Comp. Endocrinol.* **157**, 288–295 (2008).

9. L. M. Romero, M. J. Dickens, N. E. Cyr, The reactive scope model – A new model integrating homeostasis, allostasis, and stress. *Horm. Behav.* **55**, 375–389 (2009).

10. A. Wang, H. H. Luan, R. Medzhitov, An evolutionary perspective on immunometabolism. *Science* **363**, eaar3932 (2019).

11. F. A. Campos, E. A. Archie, L. R. Gesquiere, J. Tung, J. Altmann, S. C. Alberts, Glucocorticoid exposure predicts survival in female baboons. *Sci. Adv.* **7**, eabf6759 (2021).

12. J. C. Beehner, T. J. Bergman, The next step for stress research in primates: To identify relationships between glucocorticoid secretion and fitness. *Horm. Behav.* **91**, 68–83 (2017).

13. L. A. Schoenle, C. Zimmer, E. T. Miller, M. N. Vitousek, Does variation in glucocorticoid concentrations predict fitness? A phylogenetic meta-analysis. *Gen. Comp. Endocrinol.* **300**, 113611 (2021).

14. R. Ethan Pride, High faecal glucocorticoid levels predict mortality in ring-tailed lemurs (*Lemur catta*). *Biol. Lett.* **1**, 60–63 (2005).

15. K. Malkoc, L. Mentesana, S. Casagrande, M. Hau, Quantifying glucocorticoid plasticity using reaction norm approaches: There still is so much to discover! *Integr. Comp. Biol.* **62**, 58–70 (2022).

16. L. A. Schoenle, C. Zimmer, M. N. Vitousek, Understanding context dependence in glucocorticoid-fitness relationships: The role of the nature of the challenge, the intensity and frequency of stressors, and life history. *Integr. Comp. Biol.* **58**, 777–789 (2018).

17. B. C. Wheeler, B. Tiddi, M. Heistermann, Competition-induced stress does not explain deceptive alarm calling in tufted capuchin monkeys. *Anim. Behav.* **93**, 49–58 (2014).

18. S. M. Castro, G. A. Sanchez-Azofeifa, H. Sato, Effect of drought on productivity in a Costa Rican tropical dry forest. *Environ. Res. Lett.* **13**, 045001 (2018).

19. L. M. Romero, U. K. Beattie, Common myths of glucocorticoid function in ecology and conservation. *J. Exp. Zool. A Ecol. Integr. Physiol.* **337**, 7–14 (2022).

20. E. Möstl, R. Palme, Hormones as indicators of stress. *Domest. Anim. Endocrinol.* **23**, 67–74 (2002).

21. J. C. Beehner, J. Alfaro, C. Allen, M. E. Benítez, T. J. Bergman, M. S. Buehler, S. C. Carrera, E. M. Chester, T. Deschner, A. Fuentes, C. M. Gault, I. Godoy, K. M. Jack, J. D. Kim, L. Kolinski, N. K. Kulick, T. Losch, J. C. Ordoñez, S. E. Perry, F. Pinto, O. T. Reilly, E. T. Johnson, M. D. Wasserman, Using an on-site laboratory for fecal steroid analysis in wild white-faced capuchins. *Gen. Comp. Endocrinol.* **329**, 114109 (2022).

22. E. Tinsley Johnson, M. E. Benítez, A. Fuentes, C. R. McLean, A. B. Norford, J. C. Ordoñez, J. C. Beehner, T. J. Bergman, High density of white-faced capuchins (*Cebus capucinus*) and habitat quality in the Taboga Forest of Costa Rica. *Am. J. Primatol.* **82**, e23096 (2020).

23. L. M. Romero, M. Wikelski, Stress physiology as a predictor of survival in Galapagos marine iguanas. *Proc. Biol. Sci.* **277**, 3157–3162 (2010).

24. M. Lohmus, L. F. Sundstrom, F. R. Moore, Non-invasive corticosterone treatment changes foraging intensity in red-eyed vireos *Vireo olivaceus*. *J. Avian Biol.* **37**, 523–526 (2006).

25. M. Cottin, A. J. J. MacIntosh, A. Kato, A. Takahashi, M. Debin, T. Raclot, Y. Ropert-Coudert, Corticosterone administration leads to a transient alteration of foraging behaviour and complexity in a diving seabird. *Mar. Ecol. Prog. Ser.* **496**, 249–262 (2014).

26. E. Challet, Y. le Maho, J. P. Robin, A. Malan, Y. Cherel, Involvement of corticosterone in the fasting-induced rise in protein utilization and locomotor activity. *Pharmacol. Biochem. Behav.* **50**, 405–412 (1995).

27. L. Michael Romero, J. C. Wingfield, *Tempests, Poxes, Predators, and People: Stress in Wild Animals and How They Cope* (Oxford Univ. Press, 2015).

28. J. D. Orkin, M. J. Montague, D. Tejada-Martinez, M. de Manuel, J. Del Campo, S. Cheves Hernandez, A. Di Fiore, C. Fontsere, J. A. Hodgson, M. C. Janiak, L. F. K. Kuderna, E. Lizano, M. P. Martin, Y. Niimura, G. H. Perry, C. S. Valverde, J. Tang, W. C. Warren, J. P. de Magalhães, S. Kawamura, T. Marquès-Bonet, R. Krawetz, A. D. Melin, The genomics of ecological flexibility, large brains, and long lives in capuchin monkeys revealed with fecalFACS. *Proc. Natl. Acad. Sci. U.S.A.* **118**, e2010632118 (2021).

29. J. D. Orkin, F. A. Campos, M. S. Myers, S. E. Cheves Hernandez, A. Guadamuz, A. D. Melin, Seasonality of the gut microbiota of free-ranging white-faced capuchins in a tropical dry forest. *ISME J.* **13**, 183–196 (2019).

30. J. F. Gogarten, L. M. Brown, C. A. Chapman, M. Cords, D. Doran-Sheehy, L. M. Fedigan, F. E. Grine, S. Perry, A. E. Pusey, E. H. M. Sterck, S. A. Wich, P. C. Wright, Seasonal mortality

patterns in non-human primates: Implications for variation in selection pressures across environments. *Evolution* **66**, 3252–3266 (2012).

31. J. D. Hadfield, A. J. Wilson, D. Garant, B. C. Sheldon, L. E. B. Kruuk, The misuse of BLUP in ecology and evolution. *Am. Nat.* **175**, 116–125 (2010).

32. B. Taborsky, S. English, T. W. Fawcett, B. Kuijper, O. Leimar, J. M. McNamara, S. Ruuskanen, C. Sandi, Towards an evolutionary theory of stress responses. *Trends Ecol. Evol.* **36**, 39–48 (2021).

33. R. M. Sapolsky, Glucocorticoids, the evolution of the stress-response, and the primate predicament. *Neurobiol. Stress* **14**, 100320 (2021).

34. G. G. Thomas, C. Le, Stress and the HPA axis: Clinical assessment and therapeutic considerations. *Standard* **9**, 1–12 (2010).

35. F. Bonier, P. R. Martin, How can we estimate natural selection on endocrine traits? Lessons from evolutionary biology. *Proc. Biol. Sci.* **283**, 20161887 (2016).

36. K. Milton, J. Giacalone, Differential effects of unusual climatic stress on capuchin (*Cebus capucinus*) and howler monkey (*Alouatta palliata*) populations on Barro Colorado Island, Panama. *Am. J. Primatol.* **76**, 249–261 (2014).

37. B. Dantzer, E. M. Swanson, Does hormonal pleiotropy shape the evolution of performance and life history traits? *Integr. Comp. Biol.* **57**, 372–384 (2017).

38. D. Nettle, M. Bateson, Adaptive developmental plasticity: What is it, how can we recognize it and when can it evolve? *Proc. Biol. Sci.* **282**, 20151005 (2015).

39. M. Hau, S. Casagrande, J. Q. Ouyang, A. T. Baugh, Glucocorticoid-mediated phenotypes in vertebrates: Multilevel variation and evolution. *Adv. Stud. Behav.* **48**, 41–115 (2016).

40. A. J. Lea, J. Altmann, S. C. Alberts, J. Tung, Developmental constraints in a wild primate. *Am. Nat.* **185**, 809–821 (2015).

41. D. Arabadzisz, R. Diaz-Heijtz, I. Knuesel, E. Weber, S. Pilloud, A. C. Dettling, J. Feldon, A. J. Law, P. J. Harrison, C. R. Pryce, Primate early life stress leads to long-term mild hippocampal decreases in corticosteroid receptor expression. *Biol. Psychiatry* **67**, 1106–1109 (2010).

42. S. J. Lupien, B. S. McEwen, M. R. Gunnar, C. Heim, Effects of stress throughout the lifespan on the brain, behaviour and cognition. *Nat. Rev. Neurosci.* **10**, 434–445 (2009).

43. M. M. Sánchez, C. O. Ladd, P. M. Plotsky, Early adverse experience as a developmental risk factor for later psychopathology: Evidence from rodent and primate models. *Dev. Psychopathol.* **13**, 419–449 (2001).

44. L. M. Romero, M. Wikelski, Corticosterone levels predict survival probabilities of Galapagos marine iguanas during El Niño events. *Proc. Natl. Acad. Sci. U.S.A.* **98**, 7366–7370 (2001).

45. J. C. Wingfield, M. Hau, P. D. Boersma, L. M. Romero, N. Hillgarth, M. Ramenofsky, P. Wrege, R. Scheibling, J. P. Kelley, B. Walker, M. Wikelski, Effects of El Niño and La Niña Southern Oscillation events on the adrenocortical responses to stress in birds of the Galapagos Islands. *Gen. Comp. Endocrinol.* **259**, 20–33 (2018).

46. J. St-Pierre, D. P. Laplante, G. Elgbeili, P. A. Dawson, S. Kildea, S. King, C. Vaillancourt, Natural disaster-related prenatal maternal stress is associated with alterations in placental glucocorticoid system: The QF2011 Queensland flood study. *Psychoneuroendocrinology* **94**, 38–48 (2018).

47. F. A. Campos, K. M. Jack, L. M. Fedigan, Climate oscillations and conservation measures regulate white-faced capuchin population growth and demography in a regenerating tropical dry forest in Costa Rica. *Biol. Conserv.* **186**, 204–213 (2015).

48. K. Shan, Y. Lin, P.-S. Chu, X. Yu, F. Song, Seasonal advance of intense tropical cyclones in a warming climate. *Nature* **623**, 83–89 (2023).

49. T. Geng, F. Jia, W. Cai, L. Wu, B. Gan, Z. Jing, S. Li, M. J. McPhaden, Increased occurrences of consecutive La Niña events under global warming. *Nature* **619**, 774–781 (2023).

50. S. Perry, J. H. Manson, *Manipulative Monkeys: The Capuchins of Lomas Barbudal* (Harvard Univ. Press, 2008).

51. G. W. Frankie, S. B. Vinson, L. E. Newstrom, J. F. Barthell, Nest site and habitat preferences of Centris bees in the Costa Rican dry forest. *Biotropica* **20**, 301–310 (1988).

52. G. W. Frankie, A. Mata, S. Bradleigh Vinson, *Biodiversity Conservation in Costa Rica: Learning the Lessons in a Seasonal Dry Forest* (University of California Press, 2004).

53. K. M. Jack, L. M. Fedigan, Female dispersal in a female-philopatric species, *Cebus capucinus*. *Behaviour* **146**, 471–497 (2009).

54. S. Perry, "Chapter 4 - The behavior of wild white-faced capuchins: Demography, life history, social relationships, and communication" in *Advances in the Study of Behavior*, H. J. Brockmann, T. J. Roper, M. Naguib, J. C. Mitani, L. W. Simmons, Eds. (Academic Press, 2012), vol. 44, pp. 135–181.

55. R Core Team, *R: A Language and Environment for Statistical Computing.* (R Foundation for Statistical Computing, Vienna, Austria, 2023); https://www.R-project.org/.

56. T. B. McKee, N. J. Doesken, J. Kleist, "The relationship of drought frequency and duration to time scales" in *Proceedings of the 8th Conference on Applied Climatology* (Boston, 1993), vol. 17, pp. 179–183.

57. L. K. Tanaka, S. K. Tanaka, Rainfall and seasonal changes in arthropod abundance on a tropical oceanic island. *Biotropica* **14**, 114–123 (1982).

58. National Integrated Drought Information System, "Monitoring drought". https://www.drought.gov/what-is-drought/monitoring-drought.

59. S. D. Carnegie, L. M. Fedigan, T. E. Ziegler, Social and environmental factors affecting fecal glucocorticoids in wild, female white-faced capuchins (*Cebus capucinus*). *Am. J. Primatol.* **73**, 861–869 (2011).

60. I. Godoy, L. Vigilant, S. E. Perry, Inbreeding risk, avoidance and costs in a group-living primate, *Cebus capucinus*. *Behav. Ecol. Sociobiol.* **70**, 1601–1611 (2016).

61. P.-C. Bürkner, Advanced Bayesian multilevel modeling with the R package brms. *R J.* **10**, 395–411 (2018).

62. L. Zou, S. Cao, A. Sanchez-Azofeifa, Evaluating the utility of various drought indices to monitor meteorological drought in tropical dry forests. *Int. J. Biometeorol.* **64**, 701–711 (2020).

63. D. N. Karger, O. Conrad, J. Böhner, T. Kawohl, H. Kreft, R. W. Soria-Auza, N. E. Zimmermann, H. P. Linder, M. Kessler, Climatologies at high resolution for the earth's land surface areas. *Sci. Data* **4**, 170122 (2017).

64. D. N. Karger, S. Lange, C. Hari, C. P. O. Reyer, N. E. Zimmermann, *CHELSA-W5E5 v1.1: W5E5 v1.0 downscaled with CHELSA v2.0* (ISIMIP Repository, 2023); https://doi.org/10.48364/ISIMIP.836809.1.

65. W. Maetens, standaRdized: R package for the calculation of standardized index values (SPI, SPEI, SSI,…) on a daily basis (Github); https://github.com/WillemMaetens/standaRdized.

66. H. Wu, M. D. Svoboda, M. J. Hayes, D. A. Wilhite, F. Wen, Appropriate application of the standardized precipitation index in arid locations and dry seasons. *Int. J. Climatol.* **27**, 65–79 (2007).
